# Fibroadipogenic Progenitors Regulate the Basal Proliferation of Satellite Cells and Homeostasis of Pharyngeal Muscles via HGF Secretion

**DOI:** 10.3389/fcell.2022.875209

**Published:** 2022-05-17

**Authors:** Eunhye Kim, Fang Wu, Danbi Lim, Christopher Zeuthen, Yiming Zhang, James Allen, Laura Muraine, Capucine Trollet, Katherine E. Vest, Hyojung J. Choo

**Affiliations:** ^1^ Department of Cell Biology, School of Medicine, Emory University, Atlanta, GA, United States; ^2^ Laboratory of Molecular Diagnostics and Cell Biology, College of Veterinary Medicine, Gyeongsang National University, Jinju, South Korea; ^3^ Sorbonne Université, Inserm, Institut de Myologie, U974, Centre de Recherche en Myologie, Paris, France; ^4^ Department of Molecular Genetics, Biochemistry and Microbiology, University of Cincinnati College of Medicine, Cincinnati, OH, United States

**Keywords:** skeletal muscle stem cells, satellite cells, pharyngeal muscle, hepatocyte growth factor, fiboradipogenic progenitors, macrophages

## Abstract

Skeletal muscle stem cells, known as satellite cells (SCs), are quiescent in normal adult limb muscles. Injury stimulates SC proliferation, differentiation, and fusion to regenerate muscle structure. In pharyngeal muscles, which are critical for swallowing foods and liquids, SCs proliferate and fuse in the absence of injury. It is unknown what factors drive increased basal activity of pharyngeal SCs. Here, we determined how niche factors influence the status of pharyngeal versus limb SCs. *In vivo*, a subset of pharyngeal SCs present features of activated SCs, including large cell size and increased mitochondrial content. In this study, we discovered that the pharyngeal muscle contains high levels of active hepatocyte growth factor (HGF), which is known to activate SCs in mice and humans. We found that fibroadipogenic progenitors (FAPs) are the major cell type providing HGF and are thus responsible for basal proliferation of SCs in pharyngeal muscles. Lastly, we confirmed the critical role of FAPs for pharyngeal muscle function and maintenance. This study gives new insights to explain the distinctive SC activity of pharyngeal muscles.

## Introduction

The pharynx is a muscular passageway of the digestive and respiratory tracts, which extends from the nasal and oral cavity to the larynx and esophagus. The pharynx contains a group of skeletal muscles that play a critical role in many vital processes, such as swallowing, breathing, and speaking. Like other craniofacial muscles, pharyngeal muscles originate from non-segmented cranial mesoderm during vertebrate embryogenesis, while trunk and limb muscles are derived from somites (Mootoosamy and Dietrich, 2002; Noden and Francis-West, 2006). These distinctive embryonic origins are associated with unique transcriptional regulatory networks in myogenic progenitor cells. For example, limb muscle development is controlled by the PAX3 transcription factor, while PITX2/TBX1 controls craniofacial muscle development (Sambasivan et al., 2011a). However, both early muscle development pathways converge to a common myogenic program that requires the expression of myogenic regulatory factors, such as MYF5, MYOD, and myogenin (Kelly et al., 2004; Harel et al., 2009; Sambasivan et al., 2009). While mature craniofacial and limb/trunk muscles are histologically very similar, they are differentially susceptible to muscular dystrophies. For example, extraocular muscles are typically spared in Duchenne muscular dystrophy (Khurana et al., 1995) but are preferentially affected by oculopharyngeal muscular dystrophy (OPMD), a late-onset genetic disorder characterized by progressive dysphagia and ptosis (Victor et al., 1962).

An important common feature of craniofacial and limb/trunk muscles is the presence of muscle-specific stem cells termed satellite cells (SCs). SCs are a heterogeneous population of progenitor cells underneath the basal lamina of muscle fibers (Mauro, 1961; Lepper et al., 2009; Sambasivan et al., 2011b) that are crucial for skeletal muscle regeneration (Montarras et al., 2005; Sambasivan et al., 2011b; Lepper et al., 2011; Murphy et al., 2011; Fry et al., 2015). Like most other adult stem cells, SCs are quiescent under basal physiological conditions. When activated by injury, increased load, or disease, SCs rapidly re-enter the G_1_ phase of the cell cycle, proliferate as myoblasts, and progress along a defined differentiation program, leading to myogenesis (Shi and Garry, 2006). The properties of SCs during regeneration have been extensively investigated using easily accessible limb muscles. Less is known about craniofacial muscle SCs, but it has been shown that SCs from pharyngeal muscles (Randolph et al., 2015) and extraocular muscles (Stuelsatz et al., 2015) contain a population of activated SCs that chronically proliferate and differentiate into myofibers in the absence of muscle damage. The increased SC activity in craniofacial muscles raises the question of whether their unique biological and physiological properties are influenced by cell intrinsic factors or by their specialized microenvironment, known as the niche. Multiple studies have demonstrated that extracellular components like collagen (Baghdadi et al., 2018), diffusible cytokines, and growth factors released from neighboring cells, such as resident or infiltrating macrophages and fibroadipogenic progenitors (FAPs) (Evano and Tajbakhsh, 2018; Helmbacher and Stricker, 2020; Theret et al., 2021), have a major influence on SC activity in limb muscles (Vishwakarma et al., 2017). FAPs are resident mesenchymal stem cells in muscle interstitium and have been reported to be activated after tissue damage (Joe et al., 2010). FAPs have been shown to support SC differentiation *in vitro* and are required for long-term homeostatic maintenance of skeletal muscle (Heredia et al., 2013; Wosczyna et al., 2019; Uezumi et al., 2021). However, it is not clear yet whether FAPs have a role in distinct properties of craniofacial SCs. In fact, very few studies have investigated how the unique niche of craniofacial muscles affects SC activity (Formicola et al., 2014).

In this study, we compared SCs from pharyngeal and gastrocnemius muscles to understand the relative contribution of cell-intrinsic and environmental factors to the elevated basal proliferation of pharyngeal SCs. We demonstrate that a subset of pharyngeal SCs resembles activated SCs with larger cell sizes and increased mitochondrial content. Additionally, we show that pharyngeal muscles contain an active form of hepatocyte growth factor (HGF), a known SC activator (Allen et al., 1995; Walker et al., 2015). We find that FAPs and CD206^+^ resident macrophages secrete HGF and FAPs provide HGF activating enzymes including tissue-type plasminogen activator (PLAT). Lastly, based on experiments performed in FAPs-ablated mice, we conclude that FAPs within the pharyngeal muscle are partially responsible for the active proliferation of pharyngeal SCs and are critical for pharyngeal muscle function. These studies provide insight into the unique properties of craniofacial SCs and the craniofacial muscle niche, which may explain the differential susceptibility of these muscles to muscular dystrophies.

## Materials and Methods

### Mice

C57BL/6J mice (Jax000664), *Pax7 Cre* ^*ERT2*^ mice (Jax017763), *Rosa* ^*tdTomato*^ (Jax007909), *Rosa-DTA* (Jax009669), *Pax3 Cre* (Jax005549), *Rosa* ^*mT/mG*^ (Jax007576), PDGFRα *Cre* ^*ERT*^ (Jax018280), and Cx3cr1 *Cre* ^*ERT2*^ (Jax020940) were purchased from Jackson Laboratories (Bar Harbor, ME). Mouse age and genotype information was used as noted in figure legends. Homozygous *Pax7 Cre* ^*ERT2*^ male mice were crossed with homozygous *Rosa* ^*flox-stop-flox-tdTomato*^ (tdTomato) to obtain *Pax7 Cre* ^*ERT2/+*^; *Rosa* ^*tdTomato/+*^ (*Pax7 Cre* ^*ERT2*^ *-tdTomato*) mice (Sambasivan et al., 2011b). To label satellite cells with red fluorescence (tdTomato), tamoxifen at 1 mg (Sigma-Aldrich, St. Louis, MO) per 10 g body weight was injected intraperitoneally once daily for 5 days. Immunostaining was used to determine the recombination efficiency in *Pax7 Cre* ^*ERT2*^ *-tdTomato* mice. tdTomato^+^ cells from pharyngeal and gastrocnemius muscles were attached to a glass slide by cytospin and immunostained with anti-Pax7 antibody to calculate efficiency (*n* = 3). Gastrocnemius muscles (GA) have 97.4 ± 2.3% recombination efficiency and pharyngeal (PH) muscles have 90.5 ± 6.6% efficiency (Supplementary Figure S1), which is consistent with a previous report (Randolph et al., 2015). Quantitative polymerase chain reaction (qPCR) was used to determine the recombination efficiency in *Pax7 Cre* ^*ERT2*^ *-DTA* and PDGFRα *Cre* ^*ERT*^-DTA mice. Experiments were performed in accordance with approved guidelines and ethical approval from Emory University’s Institutional Animal Care and Use Committee [PROTO20170233 (Choo)] and in compliance with the National Institutes of Health.

### Muscle Tissue Preparation

Pharyngeal tissue dissection was performed as described previously with small modifications (Randolph et al., 2015). Briefly, histologic sections included pharyngeal tissues that extend from the soft palate caudally to the cranial aspects of the trachea and esophagus. Gastrocnemius muscles were used as control limb muscles. Muscle tissues were frozen in Tissue Freezing Medium (Triangle Biomedical Sciences, Durham, NC) and stored at −80°C. Cross-sections were prepared longitudinally to capture circular outside constrictor muscles, including cricopharyngeal muscles. Tissue cross-sections of 10 μm thickness were collected every 100 μm using a Leica CM1850 cryostat.

For 3- or 7-day injured muscle tissues, mice were anesthetized with isoflurane. The injury was induced in tibialis anterior (TA) muscles of anesthetized mice by injection of 25 μl of 1.2% BaCl_2_ using a Hamilton syringe (Cat# 8425 Hamilton, Reno, NV). For analgesia, mice were injected subcutaneously with 1 mg/kg buprenorphine 72-h sustained-release (SR) before muscle injury. Muscles were collected 3 or 7 days after injury.

Human muscle tissues were obtained from autopsy and biopsy. Tibialis anterior and cricopharyngeus muscles were dissected by autopsy from donated bodies within 24 h postmortem as a part of the Emory Body Donor Program. Human biopsies (quadriceps and cricopharyngeal muscle) were obtained from the Myobank-AFM, a tissue bank affiliated to EuroBioBank and with national authorization to distribute human tissues (authorization AC-2019-3502 from the French Ministry of Research) with consent from the patient. Control cricopharyngeal muscles were obtained during otolaryngological surgery after informed consent in accordance with the French legislation on ethical rules.

### Fluorescence-Activated Cell Sorting

To obtain purified satellite cells (SCs), primary cells were isolated as described previously with small modifications (Choo et al., 2016). Briefly, dissected pharyngeal and gastrocnemius muscles were minced and digested using 0.2% collagenase II (Gibco, Carlsbad, CA) and 2.5 U/ml Dispase II (Gibco, Carlsbad, CA) in Dulbecco’s modified Eagle’s medium (DMEM) at 37°C while shaking at 80 rpm for 90 min. Digested muscles were then rinsed with the same volume of Ham’s F10 media (11550043, Thermo Fisher Scientific, Waltham, MA) containing 20% FBS (F0926, Sigma, St. Louis, MO) and 100 µg/ml penicillin/streptomycin (P/S) (15140122, Thermo Fisher Scientific, Waltham, MA). Mononucleated cells were collected using a 70 µm cell strainer (Thermo Fisher Scientific, Waltham, MA). To facilitate rapid isolation of pure pharyngeal and hind limb SCs, we used a lineage labeling strategy where Pax7 positive SCs are marked with red fluorescence, tdTomato, upon tamoxifen-mediated Cre recombinase activation. Fluorescence-activated cell sorting (FACS) was performed using a BD FACSAria II cell sorter (Becton-Dickinson, Franklin Lakes, NJ) at the Emory University School of Medicine Core Facility for Flow Cytometry. Analyses of flow cytometry data were performed using FACSDiva (BD version 8.0.1) and FCS Express 6 Flow. FACS-purified SCs were plated at 500 cells per well in a 48-well plate coated with Matrigel (354277; Corning Life Sciences, New York, NY) and cultured for 5 days in Ham’s F10 media containing 20% FBS and 25 ng/ml basic fibroblast growth factor (100-18B, PeproTech, Cranbury, NJ).

To sort CD206^+^ macrophages, mononuclated cells were isolated as described above and labeled with surface proteins including 1:400 CD31-PE (clone 390; eBiosciences, San Diego, CA), 1:400 CD45-PE-Cy7 (clone 30-F11; BD Biosciences, San Jose, CA), 1:100 CD11b-FITC (clone M1/70; BD Biosciences, Vancouver, Canada), and 1:100 CD206-APC (clone C068C2; BioLegend, San Diego, CA). CD206^+^ macrophages were collected according to the following sorting criteria, CD31^-^/CD45^+^/CD11b^+^/CD206^+^ using a BD FACSAria II cell sorter (Becton-Dickinson, Franklin Lakes, NJ) at the Emory University School of Medicine Core Facility for Flow Cytometry. After sorting, cells were centrifuged at 900 g for 10 min at 4°C. Cell pellets were snap-frozen by liquid nitrogen and stored in an ultra-low freezer for gene expression analysis.

### Cytospin

To attach isolated satellite cells on glass slides for cell size analysis and immunostaining, we put cells on cytofunnels (5991040, Thermo Fisher Scientific, Waltham, MA), assembled them with slide glasses filter cards, and spun down at 1,300 rpm for 3 min (Cytospin 3, Shandon). After centrifugation, cells were fixed with 2% paraformaldehyde for 10 min and washed with PBS 3 times before immunostaining.

### *In Vivo* Cell Proliferation Assays by Flow Cytometry

To compare the proliferative abilities of SCs in pharyngeal and hindlimb muscles *in vivo*, Bromo-2′-deoxyuridine (BrdU) assays were performed. Three-month-old male mice were injected with 10 µg BrdU (Sigma-Aldrich, St. Louis, MO)/g body weight intraperitoneally every 12 h for 2 days before sacrifice. Muscles were dissected and digested as described above. To assess proliferation, isolated mononucleated cells from pharyngeal or gastrocnemius muscles were immunostained with the following antibodies: 1:400 CD31-PE (clone 390; eBiosciences, San Diego, CA), 1:400 CD45-PE (clone 30-F11; BD Biosciences, San Jose, CA), 1:4000 Sca-1-PE-Cy7 (clone D7; BD Biosciences, Vancouver, Canada), and 1:20 a7-integrin-APC (FAB3518A; R&D SYSTEMS, Minneapolis, MN). Subsequently, cells were labeled for BrdU using a BrdU flow kit (BD biosciences, Vancouver, Canada), and proliferating SCs and FAPs were collected according to the following sorting criteria, CD31^-^/CD45^-^/Sca1^-^/Intergrin7α^+^/BrdU^+^ and CD31^-^/CD45^-^/Sca1^+^/BrdU^+^, respectively using BD FACS LSR II or BD FACSymphony A3 flow cytometer (flow rate: 300–1,000 event/sec). Single cells were selected by FSC-A vs. FSC-H and then selected by SSC-A vs. SSC-H (Supplementary Figure S2A). Gating for SCs, FAPs, BrdU was drawn by FMO samples (Supplementary Figures S2B,C). Cells from gastrocnemius muscles were used as limb muscle controls.

### Culture of Myogenic Progenitor Cells

Mononucleated cells were isolated from the hindlimb muscles as described previously (Jansen and Pavlath, 2006). Briefly, dissected pharyngeal and gastrocnemius muscles were minced and digested using 0.1% Pronase (EMD Millipore, Billerica, MA) and 25 mM HEPES in Dulbecco’s modified Eagle’s medium (DMEM) at 37°C while magnetic bar-stirring at 150 rpm for 60 min. Digested muscles were pelleted by centrifugation (1,000 g for 3 min) then rinsed with the same volume of DMEM media containing 10% FBS (F0926, Sigma, St. Louis, MO) and 100 µg/ml penicillin/streptomycin (P/S) (15140122, Thermo Fisher Scientific, Waltham, MA) for trituration using 25 ml serological pipette. Mononucleated cells were collected using a 70 µm cell strainer (Thermo Fisher Scientific, Waltham, MA). Isolated satellite cells were cultured in Ham’s F-10 (11550043, Thermo Fisher Scientific, Waltham, MA), 20% FBS, 5 ng/ml basic fibroblast growth factor (100-18B, PeproTech, Cranbury, NJ), and P/S on plates coated with Collagen I (A1064402, Gibco, Thermo Fisher Scientific, Waltham, MA) for 3 or 5 days inside an incubator with *5*% CO_2_ and atmospheric O_2_ concentration (−20%) (HERAcell VIOS 160i CO_2_ incubator, Thermo Fisher Scientific, Waltham, MA). If necessary, we performed pre-plating to maintain pure MPC culture (Gharaibeh et al., 2008). Briefly, trypsinized MPCs were incubated with culture media in regular tissue culture dishes without collagen coating for 45 min to allow the settling of rapidly adhering cells. Then medium with non-adhering cells including MPCs, was moved to collagen-coated tissues culture dishes. Culture medium was exchanged every other day. MPCs with fewer than 10 passages were used for experiments.

### Fusion Index and Nuclear Number Analysis

For the fusion assay of freshly isolated SCs, sorted SCs were cultured for 10 days to induce spontaneous differentiation (Stuelsatz et al., 2015). For the fusion assay of MPCs, pharyngeal and gastrocnemius MPCs were seeded at low density (5 × 10^3^ cells/cm^2^) on Collagen I-coated plates to prevent cell-cell contact and differentiated for 2 days. Then, we counted and seeded cells at high density (7.5 × 10^4^ cells/cm^2^) to initiate prompt fusion and further differentiated them for additional 2 days. At the end of differentiation, cells were fixed in 2% formaldehyde in PBS for 10 min at room temperature and stained with Phalloidin-iFluor 594 (ab176757; Abcam, United Kingdom) for 30 min at room temperature. Nuclei were then stained with 4′,6-diamidino-2-phenylindole (DAPI), and cells were mounted with Vectashield (Vector Labs, Burlingame, CA). Myoblast fusion was quantified by counting the number of myonuclei in myotubes, which were defined as containing two or more nuclei. Fusion index was calculated as the percentage of myonuclear number relative to the total number of nuclei in the images. Diameters of each myotube were measured at three points (1/4, 2/4, and 3/4 of the length) of a myotube and averaged for each myotube. We collected 10 images from random fields of view for each line.

### MitoTracker Staining

Pharyngeal and gastrocnemius muscles were dissected from *Pax7 Cre* ^*ERT*^ *-tdTomato* mice, digested into mononuclear cells, and sorted in culture media (20% FBS in F-10 media with 1% P/S) using flow cytometry. Isolated cells were incubated with 50 nM MitoTracker^®^ Green FM (M-7514; Life Technologies, Grand Island, NY) at 37°C for 30 min. The cells were washed twice in 2% FBS in HBSS buffer and observed by fluorescence microscopy (Revolve Echo, A Bico company, San Diego, CA). All images were taken on a Revolve Echo widefield fluorescence microscope using a x20 objective (UPlanFL N, Olympus) and 5 MP CMOS Monochrome Camera. To quantify SCs with high or low MitoTracker Green signal, we used flow cytometry (BD FACS LSR II flow cytometry). First, we gated tdTomato expressing cells, then we confirmed tdTomato population was clustered as a population in parent FSC-A vs. SSC-A scatter plot. We examined the tdTomato^+^ population’s mean value of FITC to detect the intensity of MitoTracker Green using FACSDiva. High MitoTracker Green gate of pharyngeal tdTomato cells was determined by MitoTracker Green histogram of gastrocnemius tdTomato cells. Overlapped plots were generated by FCS Express 6 Flow.

### Pharyngeal Mononucleated Cell Isolation by Magnetic-Activated Cell Sorting

Mononucleated cells were isolated from pharyngeal muscles using 0.2% collagenase II (Gibco, Carlsbad, CA) and 2.5 U/ml Dispase II (Gibco, Carlsbad, CA) in Dulbecco’s modified Eagle’s medium (DMEM) at 37°C while shaking at 80 rpm for 90 min. Cells were incubated for 1 min with ammonium-chloride-potassium (ACK) buffer to lyse red blood cells. Cells were washed with 2% BSA in PBS and labeled with biotin-CD31 antibodies (130-101-955; Miltenyi Biotec, Auburn, CA) (Supplementary Figure S3A). CD31^+^ cells were isolated with magnetic streptavidin-coated microbeads and a magnetic column. Magnetic bound CD31^+^ cells were collected by flushing the magnetic column with 1 ml of PBS. Subsequently, CD31^−^ cells (unbound cells from previous MACS) were labeled with Biotin-CD45 antibodies (130-101-952; Miltenyi Biotec), and CD45^+^ cells with anti-biotin microbeads were purified. Magnetic bound CD45^+^ cells were collected by flushing the magnetic column with 1 ml of PBS. Unbound cells from both purifications were defined as CD31^−^/CD45^−^ cells. Lastly, CD31^−^/CD45^−^ cells (unbounded cells from previous MACS) were labeled with anti-PDGFRα microbeads (130-101-547; Miltenyi Biotec) using a magnetic column. Magnetic bound PDGFRα^+^ cells were collected by flushing the magnetic column with 1 ml of PBS. Bound cells from both purifications were defined as CD31^−^/CD45^−^/PDGFRα^+^ cells. Unbound cells from previous purifications were defined as CD31^−^/CD45^−^/PDGFRα^−^ cells. Cell pellets were snap-frozen by liquid nitrogen and stored in an ultra-low freezer for gene expression analysis.

### Culture of Fibroadipogenic Progenitors

CD31^−^/CD45^−^/PDGFRα^+^ cells were collected by MACS as above and cultured in DMEM, 10% FBS, 2.5 ng/ml basic fibroblast growth factor, and P/S on Matrigel-coated plates inside an incubator with 5% CO_2_ and atmospheric O_2_ concentration (−20%) (HERAcell VIOS 160i CO_2_ incubator, Thermo Fisher Scientific, Waltham, MA) until 70% confluency. Culture medium was exchanged every other day. FAPs passeged 2 or 3 times were used to obtain media for ELISA of HGF.

### ELISA of Hepatocyte Growth Factor

Media were collected from dishes of 70% confluent FAPs isolated from either gastrocnemius or pharyngeal muscles. FAPs number was counted by hemocytometer to normalize HGF concentration after media collection. Fresh FAP growth medium was used as blank. We followed the user instructions for murine pre-coated HGF ELISA kit (BGK08048; PeproTech, Rocky Hill, NJ) to measure HGF concentration in FAPs media.

### Gene Expression Analysis by Real-Time Quantitative Polymerase Chain Reaction

Pharyngeal and gastrocnemius muscles and sorted cells from both muscles were analyzed for the expression of related markers by quantitative reverse transcriptase PCR (qPCR). Total RNA from samples was extracted using Trizol (Invitrogen, Carlsbad, CA) according to the manufacturer’s instructions. Isolated RNA (250 ng) was reverse transcribed into complementary DNA (cDNA) using random hexamers and M-MLV reverse transcriptase (Invitrogen, Carlsbad, CA) and analyzed by real-time qPCR. Amplification of cDNA was performed using Power SYBR^®^ Green Master Mix (Applied Biosystems, Waltham, MA) and 2.5 μM of each primer. All primer sequences are listed in Supplementary Table S1. PCR reactions were performed for 35 cycles under the following conditions: denaturation at 95°C for 15 s and annealing + extension at 60°C for 1 min. Quantitative levels for all genes were normalized to endogenous *Hprt* expression for mouse and *RPLP0* expression for human except the SCs depletion experiment (*Pax7* gene expressions were normalized to *Acta1* gene expression). Fold change of gene expression was determined using the ∆∆Ct method (Livak and Schmittgen, 2001).

### Immunoblotting

Pharyngeal, gastrocnemius, and 3 day-injured tibialis anterior mouse muscles were homogenized by Dounce homogenizer with 500 μl of lysis buffer (20 mM Tris, pH 7.5, 150 mM NaCl, 0.1% Triton X-100) with protease and phosphatase inhibitors (Roche, Laval, Canada) (Sisson et al., 2009). Muscle lysates were incubated with lysis buffer for 1 h and centrifuged at 5,000 g for 10 min to remove tissues debris. Mouse HGF standard protein (50038-MNAH) was purchased from Sino Biological United States. Supernatants were collected and separated on SDS-polyacrylamide gel electrophoresis gels (4%–15% Mini-Protean TGX Stain-free gel, 4568086, Bio-rad, Hercules, CA) and transferred to nitrocellulose membrane (Bio-rad, Hercules, CA). The membranes were blocked in 5% non-fat dry milk for 1 h and incubated with primary antibodies (Supplementary Table S2) against HGF and GAPDH overnight. After washing, the membranes were incubated with horseradish peroxidase (HRP)-conjugated secondary antibodies {Donkey anti-mouse IgG-HRP [Jackson ImmunoResearch (715-035-15)] or Goat anti-Rabbit IgG-HRP [Bio Rad (170-6515)]} diluted 1:5000 for 1 h. Protein bands were detected using an enhanced chemiluminescence substrate kit (Thermo Fisher Scientific, Waltham, MA), and band intensity was measured by ImageJ.

### Immunohistochemistry/Immunofluorescence

Immunohistochemistry/immunofluorescence was performed as follows: cryosections were incubated with blocking buffer (5% goat serum, 5% donkey serum, 0.5% BSA, 0.25% Triton-X 100 in PBS) for 1 h and labeled with primary antibodies (Supplementary Table S2) or isotype controls overnight at 4°C in blocking buffer. The following day, sections were washed three times with washing buffer (0.2% Tween-20 in PBS) and incubated with fluorescence probe-conjugated secondary antibodies for 1 h at room temperature. We used mannose receptor-1 (CD206) as a marker for resident macrophages and platelet-derived growth factor receptor *α* (PDGFRα) as a marker for FAPs. The TSA Green kit (Tyramide Signal Amplification; Perkin Elmer, Waltham, MA) was used for CD206 and PFGFRα staining after 1 h of incubation with biotinylated donkey-anti-rabbit F(ab′)2 IgG fragments (2.5 µg/ml) to enhance the immunostaining signal. Nuclei were then stained with DAPI (1 µg/ml) and mounted using Vectashield (Vector Labs, Burlingame, CA). All images were taken on a Revolve Echo widefield fluorescence microscope using a x10 (PlanC N, Olympus) or a x20 objective (UPlanFL N, Olympus) and 5 MP CMOS Monochrome Camera.

### Behavior Measurement

Each mouse was transferred from its home cage into a new cage for single housing. We weighed a water bottle (a sipper tube with 50 ml conical tube) and dry food (Laboratory Rodent Diet 5001) and placed them in each cage. Water and food consumption and body weight for each mouse were measured at the same time every day for 4 days. On the 4th day, water was removed from the mice cage for 16 h (Overnight). After 16 h have elapsed, we reintroduced the water one at a time for each mouse and recorded the lick episodes about 30 s upon the reintroduction of water. Using the IMovie app, we changed the video play speed to 0.1X and counted the tongue protrusions/second for each mouse.

### Statistical Analyses

Statistical analysis was performed using Prism 9.0. Results are expressed as the mean ± standard error of the mean (SEM). Experiments were repeated at least three times unless a different number of repeats is stated in the legend. Statistical testing was performed using the unpaired student’s t-test or ANOVA if the data was normaly distributed by passing normality test (Shapiro-Wilk test and/or Kolmogorov-Smirnov test). If data is not normally distributed, we used non-parametric test, such as Mann-Whitney test for comparing 2 samples and Kruskal-Wallis test for comparing three or more samples. *p* < 0.05 was considered statistically significant. Statistical method, *p*-values, and sample numbers are indicated in the figure legends.

## Results

### A Subset of Pharyngeal Satellite Cells Presents Features of Activated Satellite Cells

We focused on laryngeal pharynx muscles, including thyropharyngeus (TP) and cricopharyngeus (CP) muscles (Supplementary Figure S4A), which are involved in several pharyngeal pathologies including cricopharyngeal spasm (Arenaz Búa et al., 2015) and oculopharyngeal muscular dystrophy (Gómez-Torres et al., 2012). Although pharyngeal muscles are not derived from Pax3^+^ myogenic progenitor as limb muscles are (Supplementary Figure S4B), both craniofacial and limb satellite cells (SCs) are distinguished by the expression of the paired-box/homeodomain transcription factor, PAX7. PAX7 is expressed during quiescence and early activation stages of SCs (Bosnakovski et al., 2008) and plays a key role in maintenance of self-renewed SCs (Oustanina et al., 2004). To investigate the SCs in pharyngeal muscles, we used a genetically engineered, tamoxifen-inducible *Pax7 Cre* ^*ERT2*^ *-tdTomato* mouse, which labels all PAX7 lineage-derived cells with red fluorescent protein (tdTomato) (Supplementary Figure S1). Ten days after tamoxifen injection, we observed tdTomato-labeled SCs in sectioned TP, CP, and gastrocnemius (GA, limb) muscles (Supplementary Figure S4C). The number of tdTomato-SCs in CP muscles was twice the number of SCs in GA and TP muscles (Supplementary Figure S4D), indicating that SC density is variable in different muscles (Keefe et al., 2015).

**FIGURE 1:**
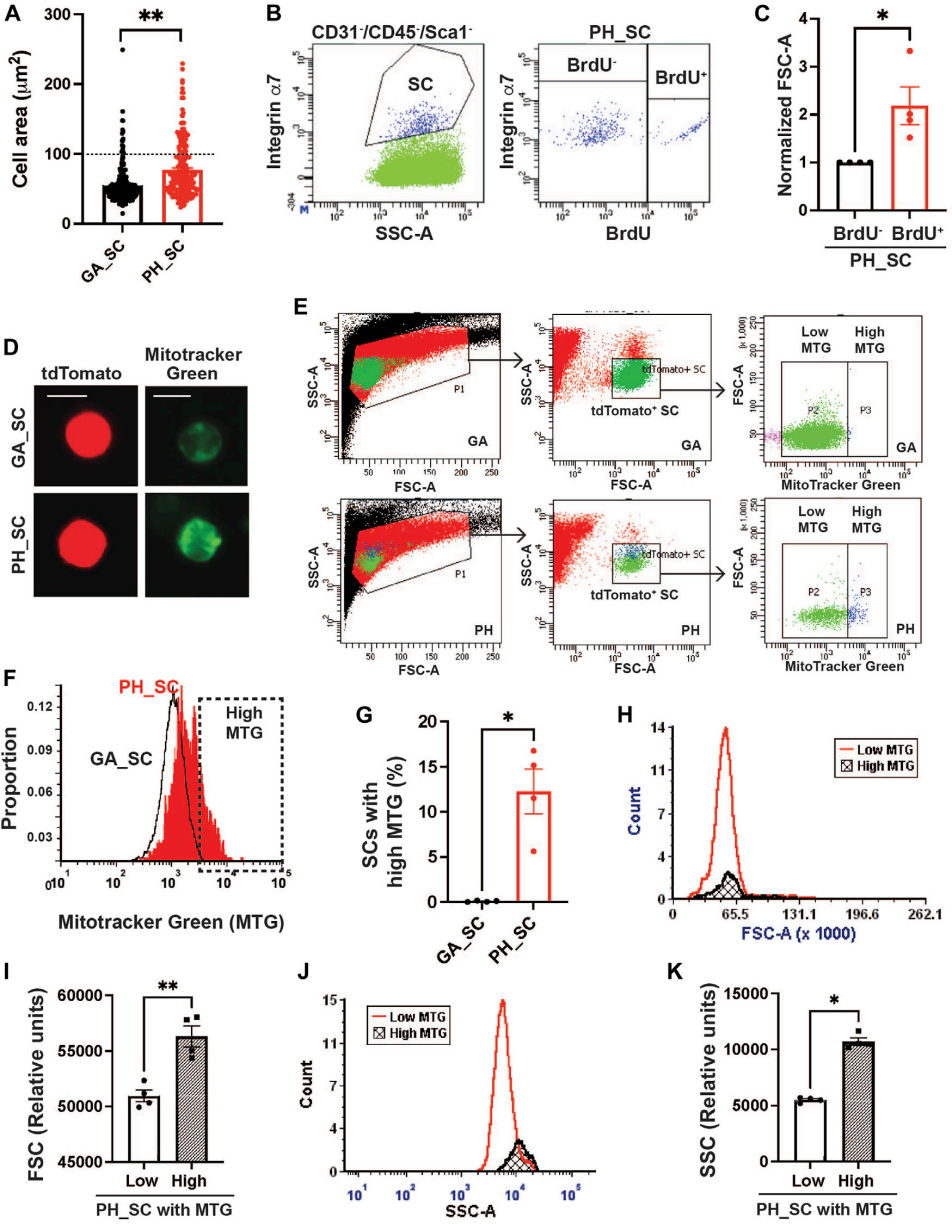
A subset of pharyngeal satellite cells are enlarged and contain abundant mitochondria. **(A)** Measured cell area (µm^2^) of pharyngeal (PH_SC) (*n* = 3, 219 cells were analyzed) and gastrocnemius SCs (GA_SC) (*n* = 3, 268 cells were analyzed) of 3 months old *Pax7 CRE* ^*ERT2*^ *-tdtomato* mice using cytospin. **(B)** Representative scatter plot showing gating strategy for pharyngeal satellite cells (SC) (Left) and BrdU^+^ pharyngeal satellite cells (Right). Satellite cells were isolated from 3 months old C57/BL6 mice. **(C)** Comparison of forward scatter (FSC-A) values between BrdU^+^ pharyngeal SC and BrdU^−^ pharyngeal SCs. FSC-A values were normalized to BrdU^−^ pharyngeal SC’s FSC. **(D)** Microscopic fluorescence images showing tdToamto expressing SCs from gastrocnemius or pharyngeal muscles mitochondria stained with MitoTracker Green. Scale bars = 10 µm. **(E)** Represntative flow gating for tdTomato^+^ GA SC and PH SC (green dots) with high (blue dots) and low MitoTracker Green intensity. **(F)** Representative flow cytometry histogram of MitoTracker Green fluorescence levels of both gastrocnemius (black line) and pharyngeal (red line) SCs. The High MTG gate indicates the SC population with high levels of MitoTracker Green. Satellite cells were isolated from 3 months Pax7CRE^ERT2^-tdtomato mice. **(G)** Percentage of SCs with high levels of MitoTracker Green (MTG) in pharyngeal (PH_SC) and gastrocnemius SCs (GA_SC). *n* = 4. **(H, J)** Representative flow cytometry histogram of forward scatter (FSC-A) or side scatter (SSC-A) comparing pharyngeal SCs (PH_SC) with low and high MitoTracker Green (MTG). **(I, K)** Quantified forward scatter (FSC-A) or side scatter (SSC-A) values for pharyngeal SCs (PH_SC) with low and high MitoTracker Green (MTG) as determined by flow cytometry. *n* = 4. Statistical significance was determined by Mann-Whitney test **(C, G, K)** or by Student’s t-test **(I)**. For all graphs, the value represents mean ± SEM. Asterisks indicate statistical significance (*p < 0.05, **p < 0.01).

When quiescent SCs are activated and begin proliferating, cell sizes are enlarged due to increased cytosolic volume and mitochondrial contents to support the energy demands of the activation (Rodgers et al., 2014). To investigate whether pharyngeal SCs exhibit characteristics similar to quiescent SCs isolated from gastrocnemius muscles, we measured the area of pharyngeal and gastrocnemius tdTomato-labeled SCs concentrated onto glass slides by cytospin (Figure 1A). The pharyngeal SCs were approximately 35% larger, with a mean area of 68.61 ± 1.611 µm^2^ compared to the gastrocnemius SC mean area of 50.89 ± 1.437 µm^2^. Interestingly, we identified two populations of pharyngeal SC that varied by size; the majority of pharyngeal SCs were similar in size to gastrocnemius SCs, but 21.5% of pharyngeal SCs were twice that size at over 100 μm^2^ (Above the dot line in Figure 1A). To confirm that the larger pharyngeal SC population represents proliferating SCs, we labeled proliferating cells using bromodeoxyuridine (BrdU). We found that BrdU^+^ pharyngeal SCs exhibit higher forward side scatter (FSC-A), indicating a larger cell size (Figures 1B,C). In addition, pharyngeal SCs contain increased mitochondria content as detected by MitoTracker Green (MTG) staining (Figure 1D) (Poot and Pierce, 1999). Quantification of MTG staining using flow cytometry (Figures 1E,F) revealed that 12% of total pharyngeal SCs contained higher mitochondrial contents (Figure 1G). Those pharyngeal SC populations with increased MTG signal were larger (increased FSC-A values) as evidenced by the slightly shifted peak in Figure 1H and average of FSC-A values (Figure 1I) and had higher granularity (increased SSC-A values) than those with low MTG signal (Figures 1J,K). To evaluate the activation status of pharyngeal SCs, we performed immunostaining using anti-MyoD antibodies on pharyngeal muscle sections of *Pax7 Cre* ^*ERT2*^ *-tdTomato* mouse (Supplementary Figure S4E). However, we did not find any MyoD^+^ SCs on pharyngeal muscle sections, which is consistent with previous microarray data indicating that pharyngeal SCs express low levels of myogenic regulatory factors including *MyoD*, *Myf5*, and *myogenin* (Randolph et al., 2015). Taken together, a subset of pharyngeal SCs is proliferating as well as presenting the features of activated SCs, such as larger cell size and increased mitochondria contents, without expression of a known SC activation marker, such as MyoD.

### Extrinsic Factors Mediate Elevated Pharyngeal Satellite Cell Proliferation

**FIGURE 2:**
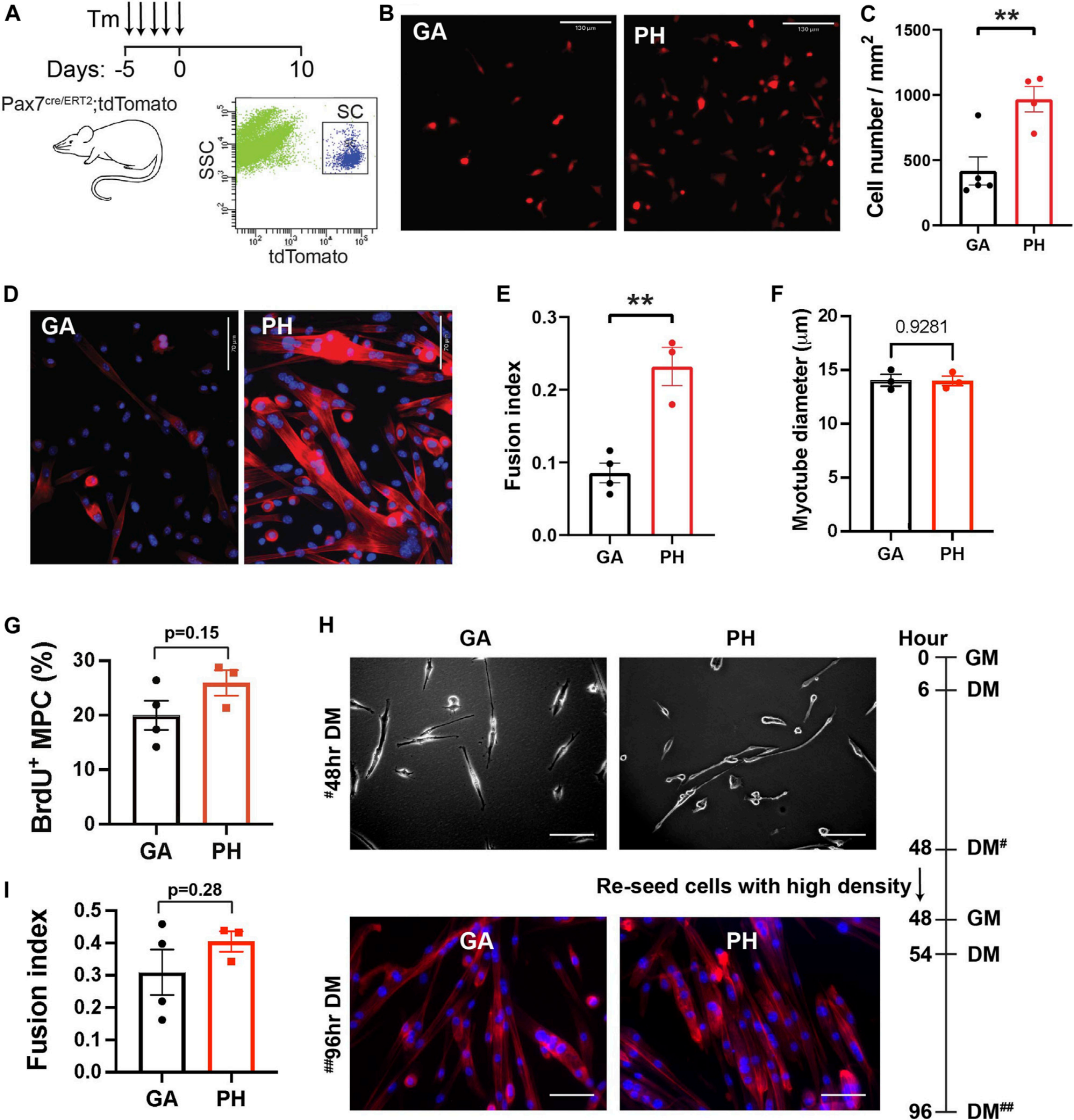
High level of *in vitro* proliferation and differentiation of freshly isolated pharyngeal satellite cells but not pharyngeal myogenic progenitor cells. **(A)** Scheme of flow cytometry gating strategy for SC isolation using 3 months old *Pax7 Cre* ^*ERT2*^ *-tdTomato* mice. **(B)** Representative image of 5-day cultured satellite cells derived from the gastrocnemius (GA) and pharyngeal (PH) muscles of 3 months *Pax7 Cre* ^*ERT2*^ *-tdTomato* mice. Scale bars = 130 µm. **(C)** Analysis of cell number/mm^2^ in 5-day cultured gastrocnemius and pharyngeal satellite cells. *n* = 4–5. **(D)** Representative Phalloidin stained image (red) of 10-day cultured satellite cells derived from the gastrocnemius and pharyngeal muscles of 3 months *Pax7 Cre* ^*ERT2*^ *-tdTomato* mice. Scale bars = 70 µm. The nucleus was stained by DAPI (blue). **(E)** Quantified fusion index at 10 days after culture. Fusion index was calculated as the percentage of total nuclei that resided in cells containing two or more nuclei. *n* = 3–4. **(F)** The diameter of myotube at 10 days after culture. *n* = 3, total of 148 GA myotubes and 353 PH myotubes were analyzed. **(G)** Percentage of BrdU^+^ myogenic progenitor cells (MPCs) in gastrocnemius and pharyngeal muscles of 3 months C57BL/6 old mice. *n* = 3. **(H)** Representative image of differentiated gastrocnemius (GA) and pharyngeal (PH) MPCs of 3 months C57BL/6 old mice. Growth medium (GM) was changed to differentiation medium (DM) 6 h after seeding. After first seeding, cells were re-seeded with high cell density in DM at 48 h^#^ with GM, and medium was changed to DM at 54 h. Scale bars = 130 µm. **(I)** Quantified fusion index at 96 h^##^. Fusion index was calculated as the percentage of total nuclei that resided in cells containing two or more nuclei. *n* = 3, total of 264 GA myotubes and 282 PH myotubes were analyzed. Statistical significance was determined by Student’s t-test **(C, E, G, I)** or by Mann-Whitney test **(F)**. For all graphs, the value represents mean ± SEM. Asterisks indicate statistical significance (**p < 0.01).

To determine whether this proliferative activity is an intrinsic property of pharyngeal SCs or is due to the pharyngeal muscle microenvironment *in vivo*, we isolated pharyngeal SCs and measured proliferation *in vitro*. SCs were sorted by tdTomato fluorescence from pharyngeal and gastrocnemius muscles of *Pax7 Cre* ^*ERT2*^ *-tdTomato* mice (Figure 2A). Equal numbers of freshly-sorted SCs from pharyngeal and gastrocnemius were seeded and then cultured for 5 days (Figure 2B). After 5 days of culture, we counted the number of cells and found twice the number of pharyngeal SCs relative to the number of gastrocnemius SCs (Figure 2C). To investigate the differentiation potential of pharyngeal SCs, we cultured freshly-sorted satellite cells for 10 days to induce spontaneous differentiation (Stuelsatz et al., 2015). The cultured pharyngeal SCs consistently exhibited increased fusion at day 10 (Figure 2D), along with an increased fusion index (Figure 2E). However, the diameter of myotubes differentiated from pharyngeal SC was similar to the myotubes from gastrocnemius SCs (Figure 2F). These results indicate that freshly isolated pharyngeal SCs still retain highly proliferative and differentiative properties *in vitro* compared to the limb satellite cells.

**FIGURE 3:**
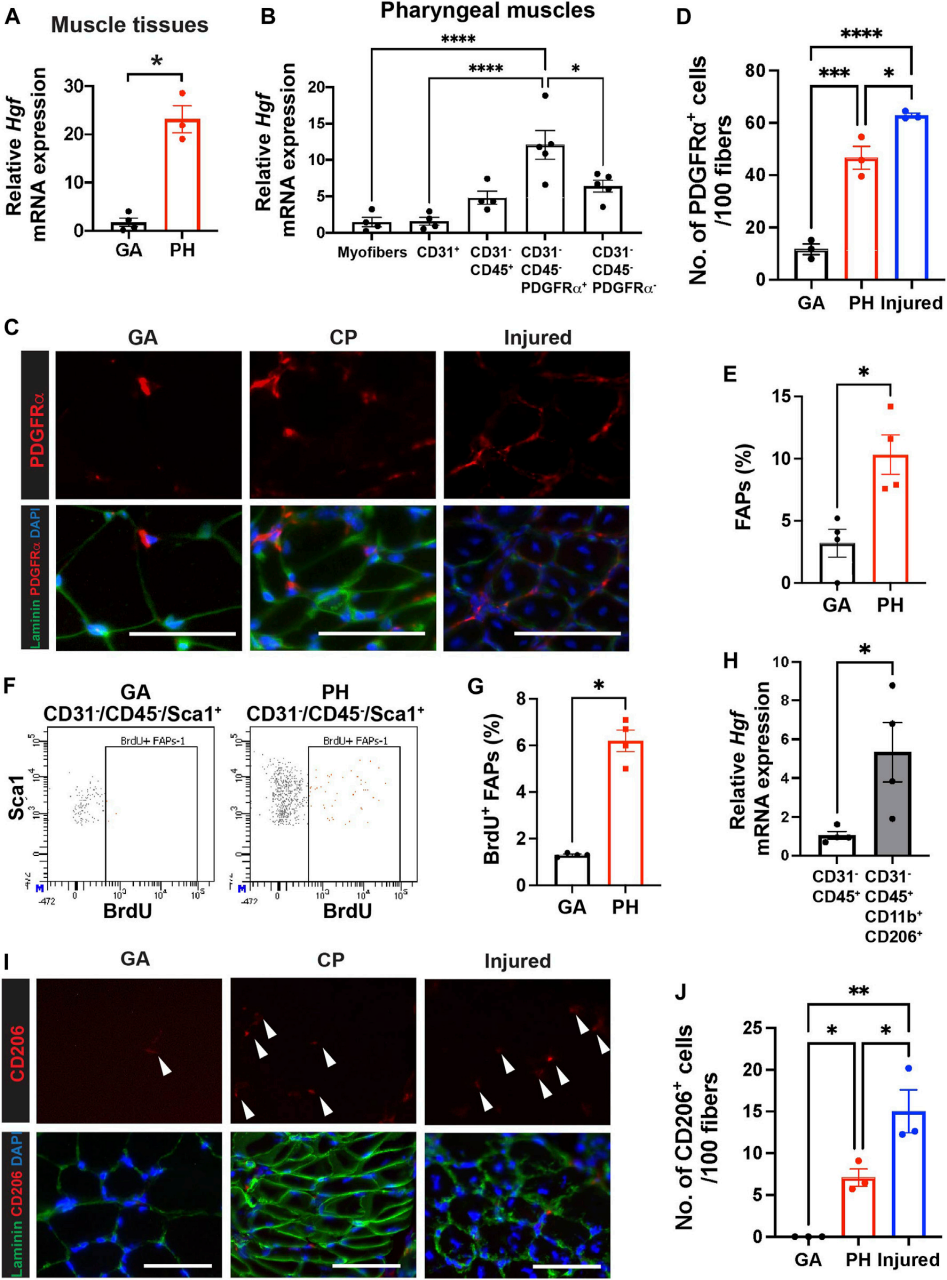
Pharyngeal muscles contain high level of HGF, FAPs and CD206^+^ macrophages. **(A)** Relative mRNA expression level of hepatocyte growth factor (*Hgf*) in gastrocnemius (GA) and pharyngeal (PH) muscles obtained from 3 month old C57BL/6 mice. *n* = 3–4. **(B)** Relative mRNA expression level of *Hgf* in myofibers and MACS-sorted CD31^+^, CD31^−^/CD45^+^, CD31^−^/CD45^−^/PDGFRα^+^ and CD31^−^/CD45^−^/PDGFRα^−^ cells obtained from muscles of 3 months old C57BL/6 mice. *n* = 4 or 5. **(C)** Representative images of PDGFRα^+^ cells in gastrocnemius (GA) and pharyngeal (PH) muscles of 3 months old C57BL/6 mice. Merged images show immunostaining with anti-PDGFRα (Red) and anti-laminin (green) antibodies and DAPI (blue). Scale bars = 50 µm. **(D)** Quantified number of PDGFRα^+^ cells per 100 myofibers. 7-day BaCl_2_-injured tibialis anterior (TA) muscles are used as a positive control. *n* = 3. **(E)** Proportion of FAPs (CD31^−^/CD45^−^/Sca1^+^) population in mononucleated cells of gastrocnemius (GA) and pharyngeal (PH) muscles by flow cytometry. **(F)** Representative dot plots of BrdU^+^ FAPs from gastrocnemius (GA) and pharyngeal (PH) muscles. **(G)** Quantified BrdU^+^ FAPs from gastrocnemius (GA) and pharyngeal (PH) muscles by flow cytometry. **(H)** The relative mRNA expression level of hepatocyte growth factor (*Hgf*) in MACS-sorted immune cells (CD31^−^/CD45^+^) and FACS-sorted CD206^+^ macrophages (CD31^−^/CD45^+^/CD11b^+^/CD206^+^) from pharyngeal muscles of 3 months old C57BL/6 mice. **(I)** Representative images of CD206^+^ cells in gastrocnemius (GA) and pharyngeal (PH) muscles of 3 months old C57/BL6 mice. Merged images show immunostaining with anti-CD206 (Red) and anti-laminin (green) antibodies and DAPI (blue). Scale bars = 50 µm. **(J)** A quantified number of CD206^+^ cells per 100 myofibers. 7-day BaCl_2_-injured tibialis anterior (TA) muscles are used as a positive control *n* = 3. Statistical significance was determined by student’s t-test **(A, E)**, by Mann-Whitney test **(G, H)**, or by 1-way ANOVA **(B, D, J)**. The value represents mean ± SEM. Asterisks indicate statistical significance (*p < 0.05, **p < 0.01, ***p < 0.001, and ****p < 0.0001).

Although *in vitro* pure SC culture experiments do not contain niche factors, prior exposure to niche factors *in vivo* may impact *ex vivo* proliferation and differentiation of SC in culture. For example, both activated SC and SC in the G-alert state, which are primed for activation by distant muscle injury (Rodgers et al., 2017), exhibit shorter first division time in the culture than freshly isolated quiescent SCs (Rodgers et al., 2014). To test whether higher proliferation and differentiation capacity found in pharyngeal SCs are intrinsic properties, we minimized the influence of prior niche factor exposure by studying pharyngeal and gastrocnemius-derived myogenic progenitor cells (MPCs) in culture. MPCs are established by growing SCs for a minimum of three passages and are more lineage-progressed than freshly isolated SCs. We seeded equal numbers of pharyngeal or gastrocnemius MPCs and cultured them for 2 days in identical conditions before performing a BrdU proliferation assay. As shown in Figure 3G, pharyngeal and gastrocnemius MPCs showed a similar level of BrdU^+^ proliferating cells. To assess differentiation capacity, we used a two-step differentiation protocol to minimize the effect of any differences in proliferation (Girardi et al., 2021). Pharyngeal and gastrocnemius MPCs were seeded at low density to prevent cell-cell contact and differentiated for 2 days (Figure 2H, top). After 48 h cells were re-seeded at high density for an additional 2 days to initiate prompt fusion (Figure 2H, bottom). We confirmed that the fusion index for pharyngeal MPCs was not significantly different from the gastrocnemius MPCs (Figure 2I). Taken together, these results suggest that the high levels of proliferation and differentiation in pharyngeal SCs are only observed in *in vivo* (Randolph et al., 2015) or in freshly isolated pharyngeal SCs *in vitro.* Therefore, although we can not exclude intrinsic factors, our data suggest that the high levels of pharyngeal SC proliferation and differentiation could be the result of the extrinsic factors in pharyngeal muscles.

### Fibroadipogenic Progenitors and Resident Macrophages are Responsible for the High Level of Hepatocyte Growth Factor Transcript in Pharyngeal Muscles

Hepatocyte growth factor (HGF) is a well-known activator of quiescent SCs upon muscle injury (Allen et al., 1995; Walker et al., 2015), so we hypothesized that HGF, which could be one of extrinsic factors, contributes to proliferation of pharyngeal SC *in vivo*. To demonstrate if HGF plays a role in a proliferating subset of SC in pharyngeal muscles, we studied the levels of *Hgf* transcript. We confirmed that the level of *Hgf* transcript is significantly increased in pharyngeal muscles compared to gastrocnemius muscles (Figure 3A). To identify the HGF secreting cells in pharyngeal muscles, we isolated pharyngeal myofibers, as well as mononucleated cells that reside in pharyngeal muscles. The mononucleated cells were further sorted into endothelial cells (CD31^+^), differentiated hematopoietic cells including macrophages (CD31^−^/CD45^+^), FAPs (CD31^−^/CD45^−^/PDGFRα^+^) (Motohashi et al., 2008), and other cell types including SCs (CD31^−^/CD45^−^/PDGFRα^−^), using MACS (Supplementary Figure S3A). We confirmed that each sorted population has high purity by probing for marker genes for each population using qPCR (Supplementary Figure S3B). The CD31^−^/CD45^−^/PDGFRα^+^ FAPs from pharyngeal muscles contained significantly higher levels of *Hgf* mRNA compared to other cell types (Figure 3B). We next investigated the number of FAPs in pharyngeal muscles by immunostaining for PDGFRα (Figure 3C). We detected a significant increase in PDGFRα^+^ cells per 100 fibers in pharyngeal muscles relative to uninjured gastrocnemius muscles, but this number was only 75% of the number of PDGFRα^+^ cells detected in 7-day injured TA muscles (Figure 3D). Using flow cytometry, we confirmed that pharyngeal FAPs (CD31^−^/CD45^−^/Sca1^+^) represent about 10% of mononucleated cells in pharyngeal muscles, which is 3 times higher than gastrocnemius FAPs content (Figure 3E). In addition, FAP proliferation (CD31^−^/CD45^−^/Sca1^+^/BrdU^+^) in pharyngeal muscles is 6 times higher than gastocenmius FAPs basal proliferation (Figures 3F,G). Taken together these results suggest that elevated HGF in pharyngeal muscles may be provided by the high numbers of FAPs within the niche.

We found *Hgf* RNA was also detected in the immune cell population (CD31^−^/CD45^+^) of uninjured pharyngeal muscles (Figure 3B). During muscle regeneration, anti-inflammatory M2 macrophages secrete HGF (Sisson et al., 2009) and HGF also promotes M2 macrophage polarity in regenerating muscles (Choi et al., 2019). Due to the absence of injury in pharyngeal muscles such as low level of infiltrating monocyte-derived Cx3cr1^+^macrophages (Arnold et al., 2007; Wang et al., 2020) in pharyngeal muscles similar to one in gastrocnemius muscles (Supplementary Figure S5), we focused tissue-resident macrophages in skeletal muscles rather than injury-induced, infiltrating macrophages (Wang et al., 2020; Dick et al., 2022). We used CD206 as a marker of muscle-resident macrophages (Wang et al., 2020) and found that sorted resident macrophages (CD31^−^/CD45^+^/CD11b^+^/CD206^+^) expressed higher levels of *Hgf* transcript compared to the total immune cell population (CD31^−^/CD45^+^) (Figure 3H). Lastly, we performed immunostaining to count CD206^+^ resident macrophages in pharyngeal sections (Figure 3I). The number of CD206^+^ cells per 100 fibers in pharyngeal muscles was 7 times higher than in uninjured gastrocnemius but was only 50% of the number of CD206^+^ cells in 7-day injured TA muscles (Figure 3J). These results indicate that pharyngeal muscles contain more FAPs and CD206^+^ macrophages than uninjured gastrocnemius muscles but less than regenerating limb muscles.

Although the CD31^−^/CD45^−^/PDGFRα^−^ SC-containing populations of pharyngeal muscles exhibit a trend of increased levels of *Hgf* mRNA relative to pharyngeal myofibers (*p* = 0.06), we found HGF transcript levels are comparable between pharyngeal and limb SCs by microarray (Randolph et al., 2015). In addition, there was no significant difference in the levels of *Hgf* mRNA between *SC*-ablated [*Pax7 Cre* ^*+/*−^ *-DTA* ^*+/+*^ TM (Tamoxifen injected)] and control [*Pax7 Cre* ^*+/*−^ *-DTA* ^*+/+*^ corn oil (CO, vehicle injected)] pharyngeal muscles (Supplementary Figure S6B). This result suggests that SCs are not a major source of elevated *Hgf* mRNA detected in pharyngeal muscles. Taken together, our data suggest that the increased number of FAPs and CD206^*+*^ macrophages are responsible for the high level of *Hgf* mRNA in pharyngeal muscles.

### Human Pharyngeal Muscles and Mouse Extraocular Muscles Contain High Levels of Hepatocyte Growth Factor mRNA and Contain Fibroadipogenic Progenitors and CD206+ Macrophages

**FIGURE 4:**
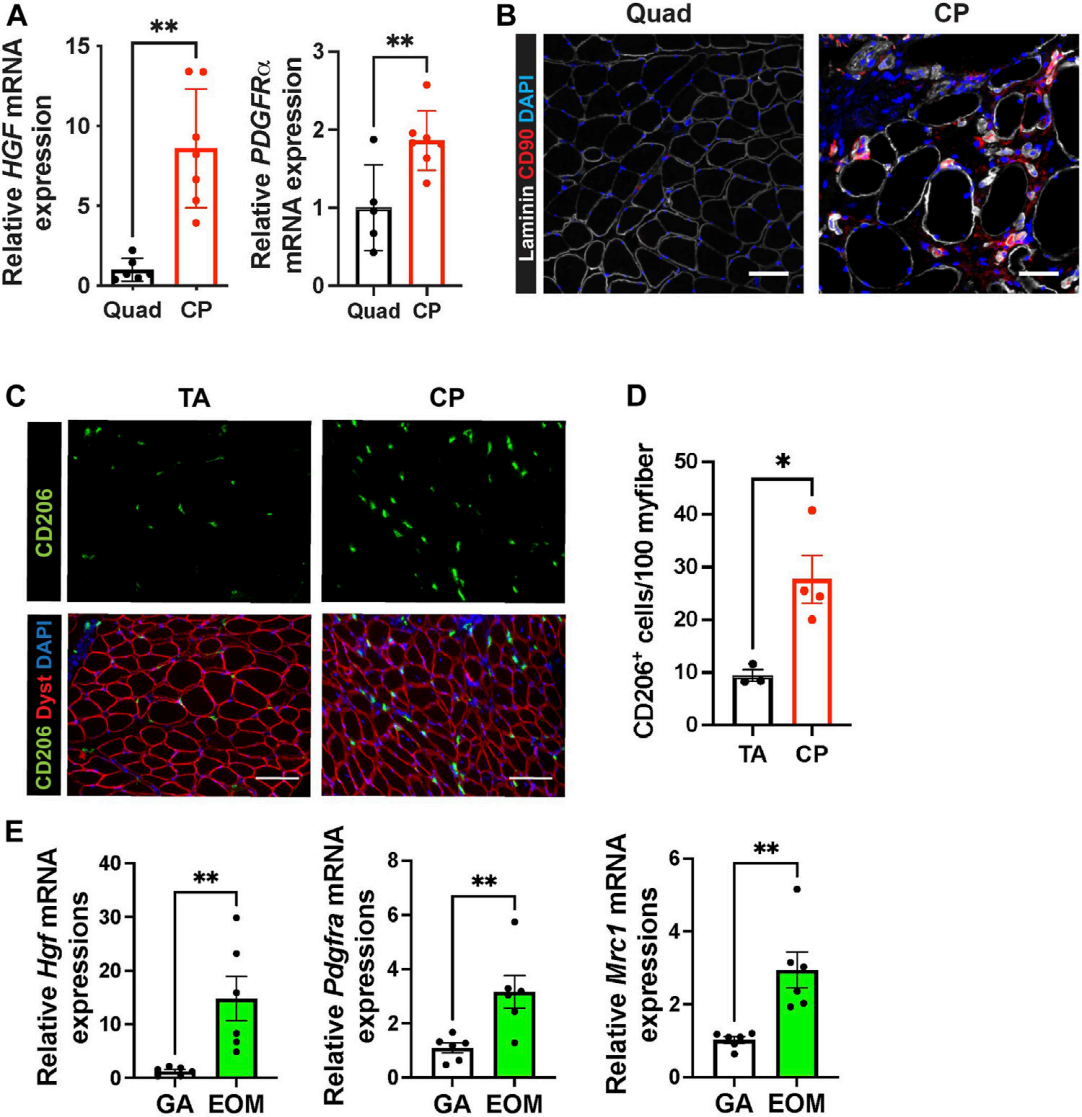
Human pharyngeal muscles and mouse extraocular muscles contain an increased level of hepatocyte growth factor, FAPs, and CD206^+^ macrophages. **(A)** Relative mRNA expression level of *HGF* and *PDGFRa* in human quadriceps (Quad) and cricopharyngeus (CP) muscles. *n* = 5–7. **(B)** Representative images of CD90^+^ FAP cells in human quadriceps (Quad) and cricopharyngeus (CP) muscles. Merged images show immunostaining with anti-CD90 (red) and anti-laminin (white) antibodies and DAPI (blue). Scale bars = 50 µm. **(C)** Representative images of CD206^+^ cells in human tibialis anterior (TA) and cricopharyngeus (CP) muscles. Merged images show immunostaining with anti-CD206 (green) and anti-dystrophin (red) antibodies and DAPI (blue). Scale bars = 130 µm. **(D)** The quantified number of CD206^+^ cells per 100 myofibers. *n* = 3–4. **(D)** Relative mRNA expression level of *Hgf*, *Pdgfra*, *Mrc1* in mouse gastrocnemius (GA) and extraocular muscles (EOM) *n* = 6. Statistical significance was determined by student’s t-test **(A, D, E)**. The value represents mean ± SEM. Asterisks indicate statistical significance (*p < 0.05 and **p < 0.01).

In mouse cricopharyngeal muscles, FAPs and residential macrophage numbers are much higher than those in gastrocnemius muscles (Figures 3D,J). To confirm that the high levels of *Hgf* RNA and relatively high numbers of FAPs and macrophages we detected in pharyngeal muscles are not exclusive to mice, we compared human pharyngeal and limb muscles. We found that human CP muscles also contain increased levels of *HGF* and *PDGFRα* mRNA (Figure 4A), increased CD90^+^ FAPs (Figure 4B) and CD206^+^ macrophages (Figures 4C,D) compared to human limb muscles. These data suggest that human pharyngeal muscles, similar to mice, provide a unique niche that could contribute to increased SC number of human cricopharyngeal muscles (Gidaro et al., 2013). Thus, the specific SC-activating niche features we detected in murine pharyngeal muscles including elevated *Hgf* provided by FAPs and resident macrophages are also true in humans. Further studies should decipher whether these features could explain pharyngeal muscle-specific pathology in humans.

Like pharyngeal muscles, mouse extraocular SCs exhibit high levels of proliferation and differentiation *in vivo* (McLoon and Wirtschafter, 2002) and *in vitro* (Stuelsatz et al., 2015). To determine if HGF provided by FAPs and CD206^+^ macrophages contributes to the proliferation and differentiation of SCs in extraocular muscles, we interrogated mouse extraocular muscles. We detected high levels of *Hgf* mRNA and markers for FAPs (*Pdgfra*) and CD206^+^ macrophages (*Mrc1*) in mouse extraocular muscles (Figure 4E). Taken together, these data suggest that elevated levels of HGF could be a common factor promoting the unusual activities of pharyngeal and extraocular SCs.

### Pharyngeal Muscles Contain Increased Levels of Active Hepatocyte Growth Factor

**FIGURE 5:**
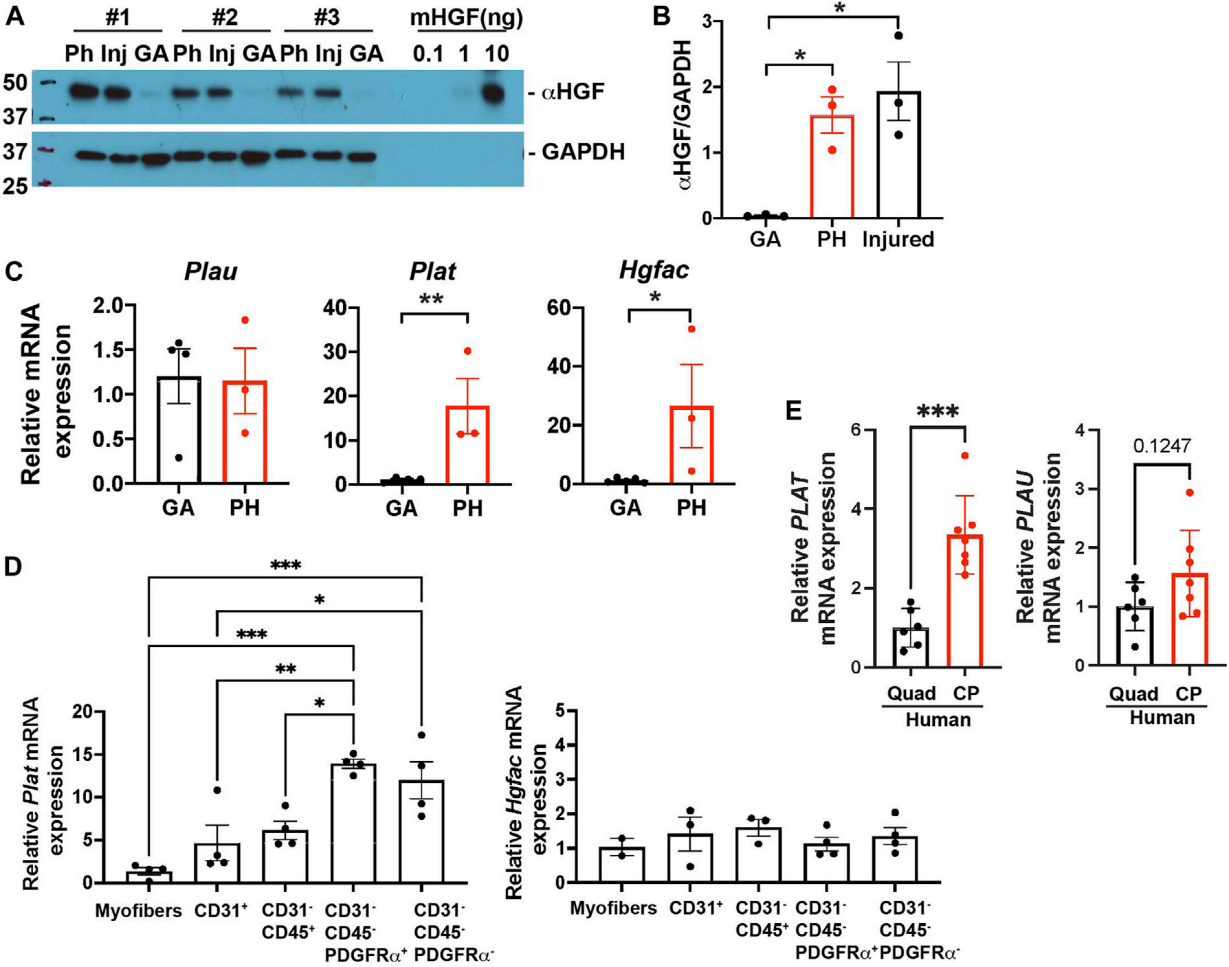
Pharyngeal muscles contain an increased level of active HGF. **(A)** Western blot analyses of the active form of HGF (αHGF) protein, and GAPDH in gastrocnemius (GA), pharyngeal (PH), and 3-day BaCl_2_-injured tibialis anterior (Inj) muscles. *n* = 3. **(B)** Normalized band intensity of the active form of HGF (αHGF) to GAPDH in gastrocnemius (GA) and pharyngeal (PH) muscles. 3-day BaCl_2_-injured tibialis anterior muscles are included as a positive control. *n* = 3. **(C)** The relative mRNA expression level of urokinase-type plasminogen activator (*Plau*), tissue-type plasminogen activator (*Plat*), and HGF activator (*Hgfac*) in gastrocnemius (GA) and pharyngeal (PH) muscles obtained from 3 months old C57BL/6 mice. *n* = 3–4. **(D)** The relative mRNA expression level of tissue-type plasminogen activator (*Plat*) and HGF activator (*Hgfac*) in myofibers and sorted CD31^+^, CD31^−^/CD45^+^, CD31^−^/CD45^−^/PDGFRα^+^, and CD31^−^/CD45^−^/PDGFRα^−^ cells obtained from muscles of 3 months old C57BL/6 mice. *n* = 3–4. Statistical significance was determined by 1-way ANOVA **(B, D)**, by student’s t-test (C except *Hgfac*, **(E)**, or by Mann-Whitney test (*Hgfac* in C). For all graphs, the value represents mean ± SEM. Asterisks indicate statistical significance (*p < 0.05, **p < 0.01, and ***p < 0.001).

**FIGURE 6:**
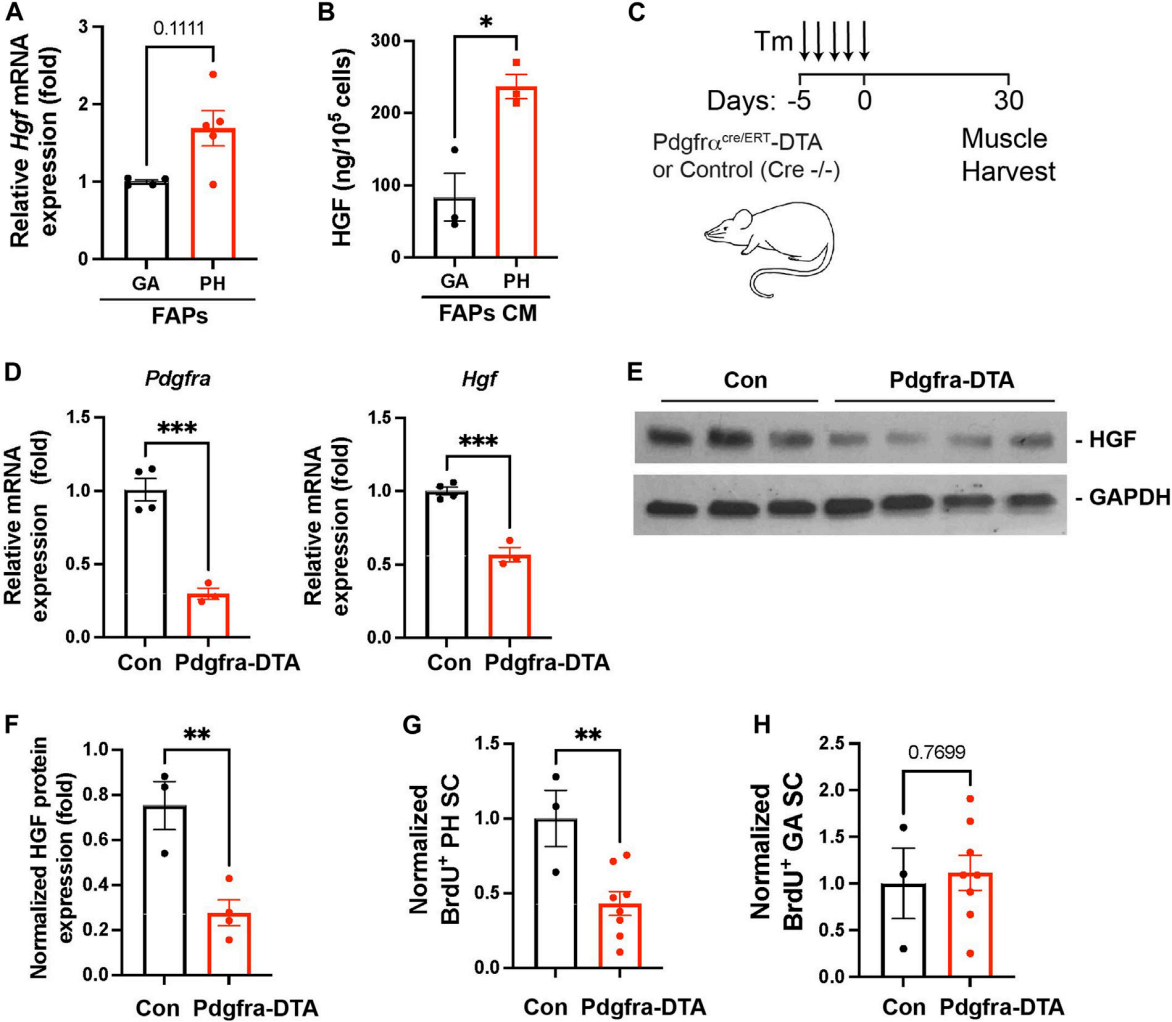
Depleted FAPs reduce HGF expression and the proliferating SC population in pharyngeal muscles. **(A)** Relative mRNA expression level hepatocyte growth factor (*Hgf*) in freshly sorted CD31^−^/CD45^−^/PDGFRα^+^ fibroadipogenic progenitors (FAPs) from gastrocnemius (GA) and pharyngeal (PH) muscles by MACS. *n* = 4–5. **(B)** Concentration of HGF in cultured medium (CM) of the gastrocnemius (GA) and pharyngeal (PH) muscles-derived fibroadipogenic progenitors (FAPs). HGF was measured by ELISA and the amount of HGF was normalized with cell number. *n* = 3. **(C)** Scheme of strategy for FAPs ablation using Pdgfrα *Cre* ^*ERT*^-DTA mouse. Tamoxifen (Tm). We used control (*Pdgfrα Cre* ^*ERT−/−*^ -*DTA*) and Pdgfra-DTA (*Pdgfrα Cre* ^*ERT+/−*^ -*DTA*) mice after 30 days of Tm injection. **(D)** The relative mRNA expression level of *Pdgfra*, and *Hgf* in pharyngeal (PH) muscles obtained from *Pdgfrα Cre* ^*ERT*^ -*DTA* mouse compared to control mice. *n* = 3–4. **(E)** Western blot analyses of the active form of HGF (αHGF) protein, and GAPDH in pharyngeal muscles of control and Pdgfra-DTA mice. *n* = 3–4. **(F)** Normalized band intensity of the active form of HGF (αHGF) to GAPDH in pharyngeal (PH) muscles. *n* = 3–4. **(G, H)** Normalized percentage of BrdU^+^ pharyngeal or gastrocnemius satellite cells (CD31^*−*^/CD45^*−*^/Sca1^*−*^/Intergrin α7^+^) isolated from Pdgfrα *Cre* ^*ERT*^ -DTA mice compared to control mice. *n* = 3 or 8. Statistical significance was determined by Mann-Whitney test **(A)** or by Student’s t-test **(B, D) (F–H)**. The value represents mean ± SEM. Asterisks indicate statistical significance (*p < 0.05, **p < 0.01, and ***p < 0.001).

HGF exists in a biologically inactive form in the extracellular matrix (ECM) of uninjured tissues (Gak et al., 1992). To actively mediate signaling, HGF is converted from an inactive precursor (pro-HGF) into a processed active form of HGF (active HGF, αHGF). To elucidate whether HGF is active in pharyngeal muscles, we used immunoblotting to measure active HGF by size as compared to mouse HGF standard proteins (Figure 6A, right side of blot). We detected activated HGF in pharyngeal and 3 day-injured TA muscles but not in gastrocnemius muscles (Figures 5A,B). Given that activation of HGF depends on cleavage by proteases, we measured multiple proteases in pharyngeal muscles. In injured muscles, urokinase plasminogen activators (PLAU) (Sisson et al., 2009) activate quiescent SCs and HGF activators (HGFAC) process HGF to promote the SC G_alert_ state in distant uninjured muscles (Rodgers et al., 2017). The tissue-type plasminogen activator PLAT activates HGF but has not been studied in the context of injured muscles. However, elevated *Plat* transcript levels were detected in pharyngeal SCs compared to limb SCs in previously published microarray data (Randolph et al., 2015). To determine which proteases likely generate active HGF in pharyngeal muscles, we measured the levels of *Plau*, *Plat*, and *Hgfac* mRNAs*.* The levels of *Plat* and *Hgfac* mRNAs were increased in whole pharyngeal muscles relative to gastrocnemius muscles (Figure 5C), indicating that HGF in pharyngeal muscles could be activated by PLAT or HGFAC rather than PLAU. In addition, *Plat* mRNA levels were increased in isolated FAPs (CD31^-^/CD45^-^/PDGFRα^+^), but *Hgfac* mRNA levels were broadly detected among the different cell types (Figure 5D). As we observed in mouse pharyngeal FAPs, human CP muscle also contain higher *PLAT* transcript levels compared to limb muscle but *PLAU* transcript levels are similar between human CP and limb muscles (Figure 5E). Taken together, these data suggest that pharyngeal FAPs are a major cell source of the HGF-activating enzyme PLAT and of HGF, which likely is responsible for the increased activation of pharyngeal SCs *in vivo*.

### Fibroadipogenic Progenitors are Responsible for Pharyngeal Satellite Cell Proliferation Through Hepatocyte Growth Factor

Our data revealed that elevated numbers of FAPs are responsible for the high levels of HGF detected in pharyngeal muscles (Figure 3B). To investigate if pharyngeal FAPs indeed secrete more HGF protein compared to gastrocnemius FAPs, we measured *Hgf* mRNA levels and secreted HGF protein levels. We found that *Hgf* mRNA levels are trended towards higher (*p* = 0.11) in FAPs derived from pharyngeal muscle compared to FAPs derived from gastrocnemius muscle (Figure 6A). Conditioned medium from pharyngeal FAPs (FAPs CM) also contained 3-fold higher levels of HGF protein compared to conditioned medium from gastrocnemius FAPs as measured by ELISA (Figure 6B). To confirm the contribution of FAPs to HGF-mediated pharyngeal SC proliferation, we generated FAPs-ablated mice using PDGFRα *Cre* ^*ERT*^-DTA mouse, which allows for the specific and conditional depletion of FAPs following tamoxifen-induced expression of diphtheria toxin (Figure 6C). Following tamoxifen treatment, pharyngeal muscles from PDGFRα *Cre* ^*ERT*^-DTA mice contained −80% less *Pdgfra* transcript in the pharyngeal muscle, confirming the effective FAPs ablation (Figure 6D left). Levels of *Hgf* mRNA (Figure 6D right) and HGF protein (Figures 6E,F) were significantly lower in FAPs-ablated pharyngeal muscles, suggesting that FAPs provide the majority of HGF in pharyngeal muscles. Furthermore, the percentage of BrdU^+^ proliferating SCs was significantly lower in FAPs-ablated pharyngeal muscles compared to the control group (Figure 6G), indicating that FAPs promote the proliferation of uninjured pharyngeal SCs. However, basal proliferation of gastrocnemius SCs was not reduced by FAPs-ablation (Figure 6H).

### Fibroadipogenic Progenitors are Critical for Pharyngeal Muscle Function and Homeostasis

**FIGURE 7:**
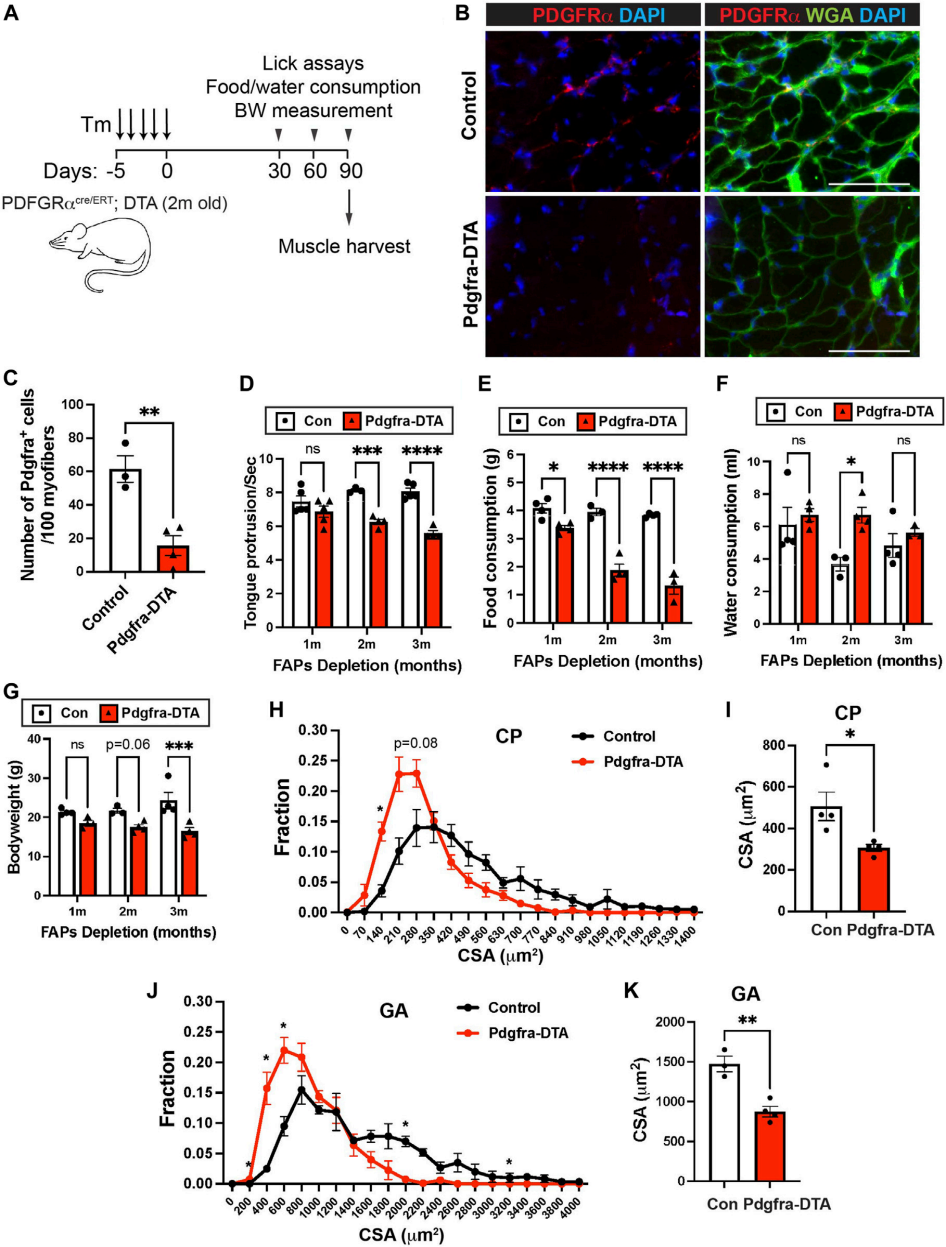
FAPs are critical for pharyngeal muscle function and maintenance. **(A)** Scheme of strategy for satellite cell ablation using Pdgfra *Cre* ^*ERT+/−*^-DTA (Pdgfra-DTA, *n* = 3–4) and Pdgfra *Cre* ^*ERT−/−*^-DTA (sex- and age-matched control, *n* = 3–4). Both groups were injected with Tamoxifen (Tm). Lick assay was performed at 1, 2, and 3 months post tamoxifen injection using the same cohort. Mice were sacrificed at 3 months of FAPs ablation for muscle harvest. **(B)** Representative images of PDGFRα^+^ cells in pharyngeal (PH) muscles of control and Pdgfra-DTA mice after 3 months of tamoxifen injection. Merged images show immunostaining with anti-PDGFRα (Red) and anti-laminin (green) antibodies and DAPI (blue). Scale bars = 70 µm. **(C)** The quantified number of PDGFRα^+^ cells per 100 myofibers. *n* = 4. **(D)** The number of tongue protrusions per second was counted when a mouse lick the water sipper using video analysis. **(E, F)** Each mouse was housed in a single cage to measure daily food (gram) and water (ml) consumption. **(G)** Bodyweight was measured at each month. **(H, J)** The cross-sectional area of CP or GA muscle fibers was measured between FAPs-ablated mice and control mice. **(I, K)** The average cross-sectional area of CP or GA muscle fibers. *n* = 3–4. Statistical significance was determined by Student’s t-test **(C, I, K)**, by multiple unpaired t-test for each size **(H, J)** or by 2-way ANOVA and Sidak’s multiple comparison test **(D–G)**. The value represents mean ± SEM. Asterisks indicate statistical significance (*p < 0.05, **p < 0.01, and ***p < 0.001).

We next measured how FAPs depletion affected pharyngeal muscle function and homeostasis. In addition to their function in HGF secretion and SC proliferation, FAPs have been shown to play a key role in muscular regeneration *via* follistatin (Iezzi et al., 2004; Mozzetta et al., 2013) and muscle maintenance *via* BMP3B (Wosczyna et al., 2019; Uezumi et al., 2021). We performed pharyngeal muscle functional assays on 6-month old tamoxifen-treated PDGFRα *Cre* ^*ERT*^-DTA (FAPs-ablated) mice at 1, 2, and 3 months after FAPs depletion (Figure 7A). Depletion of PDGFRα^+^ cells was confirmed by immunofluorescence staining using PDGFRα antibodies on pharyngeal muscle sections (Figures 7B,C). FAPs-ablated mice showed significantly reduced lick rates at both 2 and 3 months after ablation (Figure 7D), suggesting they have reduced liquid swallowing ability. Along with a reduced lick rate, food consumption was significantly decreased in FAPs-ablated mice at all time points measured (Figure 7E). Water consumption was increased after 2 months of FAPs ablation (Figure 7F), which may represent compensatory consumption due to the reduced food intake. As a consequence, FAP-ablated mice showed decreased body weight (Figure 7G). Consistent with the functional deficits, the pharyngeal muscle cross-sectional area was significantly decreased in FAPs ablated mice (Figures 7H,I). In addition to loss of the established BMP3B-mediated mechanism of FAPs-regulated muscle homeostasis and neuromuscular integrity (Uezumi et al., 2021), the reduced food intake in FAP-ablated mice likely contributed to further limb muscle loss after FAPs depletion (Figures 7J,K) (Wosczyna et al., 2019; Uezumi et al., 2021). Although we detected reduced SCs proliferation after FAPs ablation (Figures 6G,H), loss of SC activity was not responsible for defective pharyngeal muscle function since SCs ablation had minimal effect on cricopharyngeal muscle size and swallowing function (Supplementary Figure S6C–G). Taken together, these results show that depletion of FAPs impairs pharyngeal muscle function and homeostasis thus demonstrating the importance of FAPs in pharyngeal muscles along with the detrimental role of FAPs in fibrotic pharyngeal muscles of OPMD patients (Bensalah et al., 2022).

## Discussion

**FIGURE 8:**
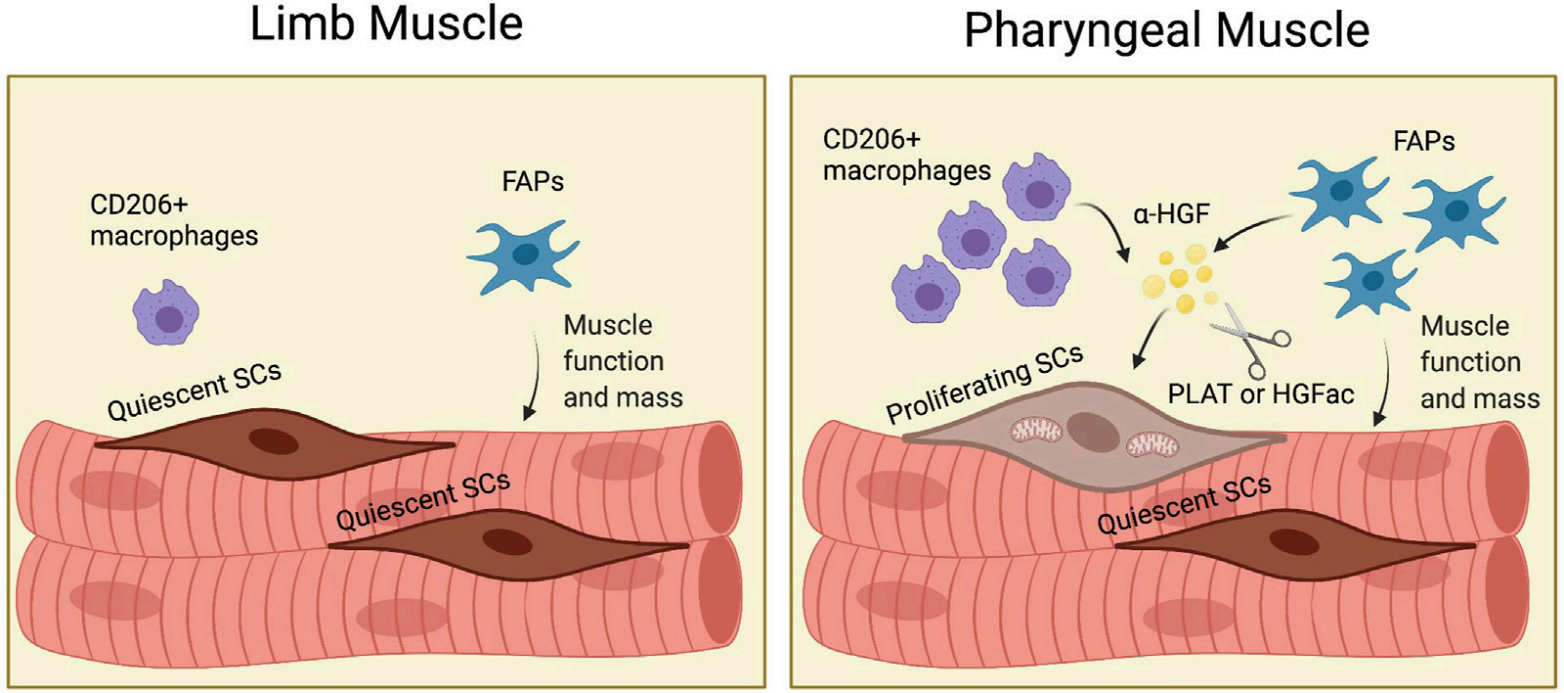
(Graphic abstract). FAPs induce proliferation of pharyngeal satellite cells by HGF and maintain pharyngeal muscle mass and function. In pharyngeal muscles, the increased number of CD206^+^ macrophages and fibroadipogenic progenitors (FAPs) secrete HGF. HGF is converted to the active form (α-HGF) by tissue-type plasminogen activator (PLAT) or HGF activator (HGFac). Therefore, FAPs mediate proliferation of SCs in pharyngeal muscles *via* HGF. In addition, FAPs maintain pharyngeal muscle mass and function. Created with BioRender.com.

Although craniofacial muscles, including pharyngeal muscles, differ from limb and trunk muscles in embryonic origin and core genetic programs (Tajbakhsh, 2009), most of what we know about SCs, FAPs, and other cell types comes from studies in limb muscles. Studies of SCs and FAPs in craniofacial muscle are difficult due to the small size of craniofacial muscles, the difficult dissection, and the lack of functional assays. We employed genetic mouse models to reveal the distinct characteristics of pharyngeal SCs and to demonstrate the role of FAPs for pharyngeal SC proliferation and pharyngeal muscle homeostasis. To explain the highly proliferative and differentiative properties of pharyngeal SCs *in vivo*, we characterized pharyngeal SCs and investigated the role of the pharyngeal muscle environment for SC proliferation. Our study provides new evidence to explain how niche factors, such as HGF, and neighboring cells including FAPs and resident macrophages, govern the unique state of craniofacial SCs (Figure 8).

### Hepatocyte Growth Factor Contributes to the Activation of Pharyngeal Satellite Cells

Neighboring cells and SC contribute to the muscle microenvironment *via* soluble factors or direct cell-to-cell contact. HGF is one such auto/paracrine factor involved in SC activation in response to muscle injury, overuse, or mechanical stretches (Tatsumi et al., 1998; Miller et al., 2000; Sheehan et al., 2000; Tatsumi, 2010). HGF is secreted into the extracellular matrix of uninjured muscles as pro-HGF and is activated by proteolysis mediated by urokinase-type plasminogen activator (PLAU) (Sisson et al., 2009) or by circulating HGF activators originating from a remote muscle injury (Rodgers et al., 2017). Cleaved HGF, in turn, activates SCs (Stoker et al., 1987; Bernet-Camard et al., 1996; Sisson et al., 2009). In pharyngeal muscles, we detected high levels of the transcripts encoding both HGF activator (*Hgfac*) and tissue-type plasminogen activator (*Plat*), which is similar (identity 32.8% and similarity 43%) to the urokinase plasminogen activator that cleaves pro-HGF (Mars et al., 1993). However, the transcript encoding urokinase plasminogen activator (*Plau*) was not increased in pharyngeal muscles. This result indicates that uninjured pharyngeal muscles use a mechanism of SC activation, that is, distinct from that of the limb. Our previous microarray data comparing pharyngeal SCs with limb SCs (Randolph et al., 2015) revealed increased levels of the *Plat* transcript, suggesting that pharyngeal SCs also contribute to pro-HGF processing. Interestingly, extraocular muscle SCs, which also proliferate and differentiate without injury, also contain high levels of *Plat* mRNA relative to limb SCs (Pacheco-Pinedo et al., 2009). In addition, extraocular muscles express high levels of *Hgf* mRNA. Thus, HGF is likely an important signal that modulates both pharyngeal and extraocular SC activity. Additional studies are needed to better define the unique mechanisms of pro-HGF processing and HGF-mediated craniofacial SC activation.

### Fibroadipogenic Progenitors Release Hepatocyte Growth Factor in Pharyngeal Muscles

Muscle is a heterogeneous tissue that contains myofibers, SCs, blood vessels, peripheral nerves, mesenchymal cells, and resident macrophages (Bentzinger et al., 2013; Tedesco et al., 2017). To identify the major source of HGF, we sorted myofibers, endothelial cells, immune cells, and others using MACS. We also ruled out the autocrine secretion of HGF from pharyngeal SCs using SC-ablated mice, which is consistent with the previous microarray analysis showing that pharyngeal SCs contain comparable *Hgf* mRNA levels to limb SCs (Randolph et al., 2015). We found the highest levels of *Hgf* mRNA in the CD31^−^/CD45^−^/PDGFRα^+^ fraction isolated by MACS. Considering that FAPs are the main population of the CD31^−^/CD45^−^/PDGFRα^+^ fraction, we focused on FAPs as the likely source of HGF in pharyngeal muscle niche. Pharyngeal muscles contain a high number of FAPs, which may be a result of pro-proliferative HGF autocrine signaling (Rodgers et al., 2017). Importantly, we discovered that FAPs are the major source of elevated HGF detected in pharyngeal muscles and are responsible for the active proliferation of pharyngeal SCs in FAPs ablation experiments. In addition to results obtained from mice, we confirmed an increased *HGF*, *PDGFRa*, and *PLAT* transcript levels and CD90^+^ FAPs in human CP muscles. The increased *HGF* levels in CP is consistent with a previous report examining several craniofacial muscles, including CP muscles from 14 cadavers (Rhee et al., 2016). These data indicate the translational potential of our data generated in mice. Like SCs, FAPs are more proliferative in pharyngeal muscles compared to limb muscles in mouse and human (Bensalah et al., 2022), which could be related to the high level of extracellular matrix (ECM) found in pharyngeal muscles compared to limb muscles in healthy humans (Gidaro et al., 2013; Bensalah et al., 2022). Future studies are needed to probe how FAPs are activated and differentiated in pharyngeal muscles, whether this activation involves interaction with CD206^+^ macrophages or HGF autocrine effects, and whether this regulation is disturbed in pharyngeal muscle-specific pathologies with excessive ECM like OPMD (Gidaro et al., 2013; Bensalah et al., 2022).

In regenerating muscles, the majority of HGF is secreted by macrophages (Sisson et al., 2009) that infiltrate the skeletal muscle niche after injury (Pillon et al., 2013). Indeed, a large number of CD206^+^ macrophages in pharyngeal muscles are detected and contribute to the high level of *Hgf* transcript, but these are likely tissue-resident rather than infiltrating macrophages because pharyngeal muscles show no signs of injury (Randolph et al., 2015) and have low levels of infiltrating Cx3cr1+ macrophages. Resident macrophages in skeletal muscles express CD206, as well as CD45, CD11b, F4/80, and CD64, and exhibit distinct transcriptional profiles when depending on whether they were isolated from limb versus diaphragm muscles (Wang et al., 2020). It is unclear why CP muscles contain high numbers of FAPs and CD206^+^ macrophages, but the pharyngeal muscle niche may play a role in the recruitment of both cells. We did not detect any signs of injury, such as clusters of immune cells, in pharyngeal muscles. However, pharyngeal muscle contains relatively high levels of centrally nucleated fibers, which we previously interpreted as a sign of continuous satellite cell fusion. Alternatively, centrally nucleated fibers may be a result of muscle turnover in pharyngeal muscles that promotes FAPs and macrophage proliferation and proliferates satellite cells. Although we did not observe embryonic myosin heavy chain^+^ myofibers, neonatal myosin heavy chain protein was detected in pharyngeal muscles using immunoblotting (Randolph et al., 2015). Given the known role of resident macrophages in skeletal and cardiac muscles (Brigitte et al., 2010; Bajpai et al., 2019; Theret et al., 2021), we speculate that CD206^+^ macrophages may regulate pharyngeal muscle homeostasis and its responses to inflammation.

### Role of Fibroadipogenic Progenitors in Craniofacial Muscles

Similar to the distinctive embryonic origins of craniofacial muscles and SCs, craniofacial FAPs are derived from cranial neural crests, while FAPs in limb/trunk muscles arise from lateral plate mesoderm-derived somites (Sefton and Kardon, 2019). Neural crest-derived muscle connective tissues, precursors of FAPs, are required for craniofacial muscle development and morphogenesis (Noden and Trainor, 2005; Rinon et al., 2007). Although less well studied, FAPs were thought to have a profound role in craniofacial muscle homeostasis and regeneration (Cheng et al., 2021). Interestingly, HGF is one of the critical factors that induce migration of muscle progenitor cells that express cMET as an HGF receptor for muscle development (Bladt et al., 1995; Dietrich et al., 1999). Given the role of muscle connective tissues during development, they influence the migration of muscle progenitor cells *via* HGF. Although we identified that FAPs are responsible for high levels of HGF and unusual SC proliferation in pharyngeal muscles, we still do not understand the functional importance of the proliferating SCs in adult pharyngeal muscles. We speculate that a subset of proliferating SCs could be influenced by HGF and other factors secreted by local FAPs. While we can not exclude intrinsic factors of SC for pharyngeal muscle homeostasis, we found satellite cell-ablated mice present normal swallowing function and pharyngeal muscle size, the role of satellite cells for pharyngeal muscles may not be critical at least in physiologic conditions. However, their distinct behaviors, such as proliferation without injury and continuous fusion to pharyngeal and extraocular muscles, should be analyzed in craniofacial muscle-specific diseases, such as oculopharyngeal muscular dystrophy (OPMD). In contrast to the dispensable role of SCs for pharyngeal muscle function, we found FAPs to be critical for pharyngeal muscle function and maintenance. Impaired pharyngeal muscle function may explain rapid loss of body weight and muscle mass following FAPs depletion in our study and previous study (Wosczyna et al., 2019; Uezumi et al., 2021). However, heterogeneous origins and a lack of specific markers are challenges in investigating craniofacial FAPs. In this study, we used a PDGFRα *Cre* ^*ERT*^-DTA mouse model to investigate the role of FAPs in the basal proliferation of SC in pharyngeal muscles. While pharyngeal SCs showed less proliferation with FAPs depletion in PDGFRα *Cre* ^*ERT*^-DTA mouse, we cannot exclude the influence of removed stromal cells in other tissues of this mouse model. Localized FAPs-depletion (Wosczyna et al., 2019) would not be feasible due to the deep location and thin structure of pharyngeal muscles that would make impossible to access with locally-injected AAV.

In conclusion, this study is the first report to identify niche/environmental factors associated with the highly proliferative features of pharyngeal SCs. While it is not clear whether the unique embryonic origins of pharyngeal muscles lead to the differences in proliferative and myogenic properties of SCs, this study demonstrates that the pharyngeal muscle niche, such as FAPs and HGF from FAPs, is capable of proliferating pharyngeal SCs without injury. Although the role of highly active SCs in pharyngeal muscle function is still ambiguous, we propose that unique properties of pharyngeal SCs and FAPs would provide useful information to understand pharyngeal muscle-specific pathologies, such as oculopharyngeal muscular dystrophy and dysphagia.

## Data Availability

The original contributions presented in the study are included in the article/Supplementary Material, further inquiries can be directed to the corresponding author.
