# Supplementary figures and images for: Fibroadipogenic Progenitors Regulate the Basal Proliferation of Satellite Cells and Homeostasis of Pharyngeal Muscles via HGF Secretion
Source: Front Cell Dev Biol. 2022 May 17;10:875209. doi: 10.3389/fcell.2022.875209 (PMC9164287; doi:10.3389/fcell.2022.875209)

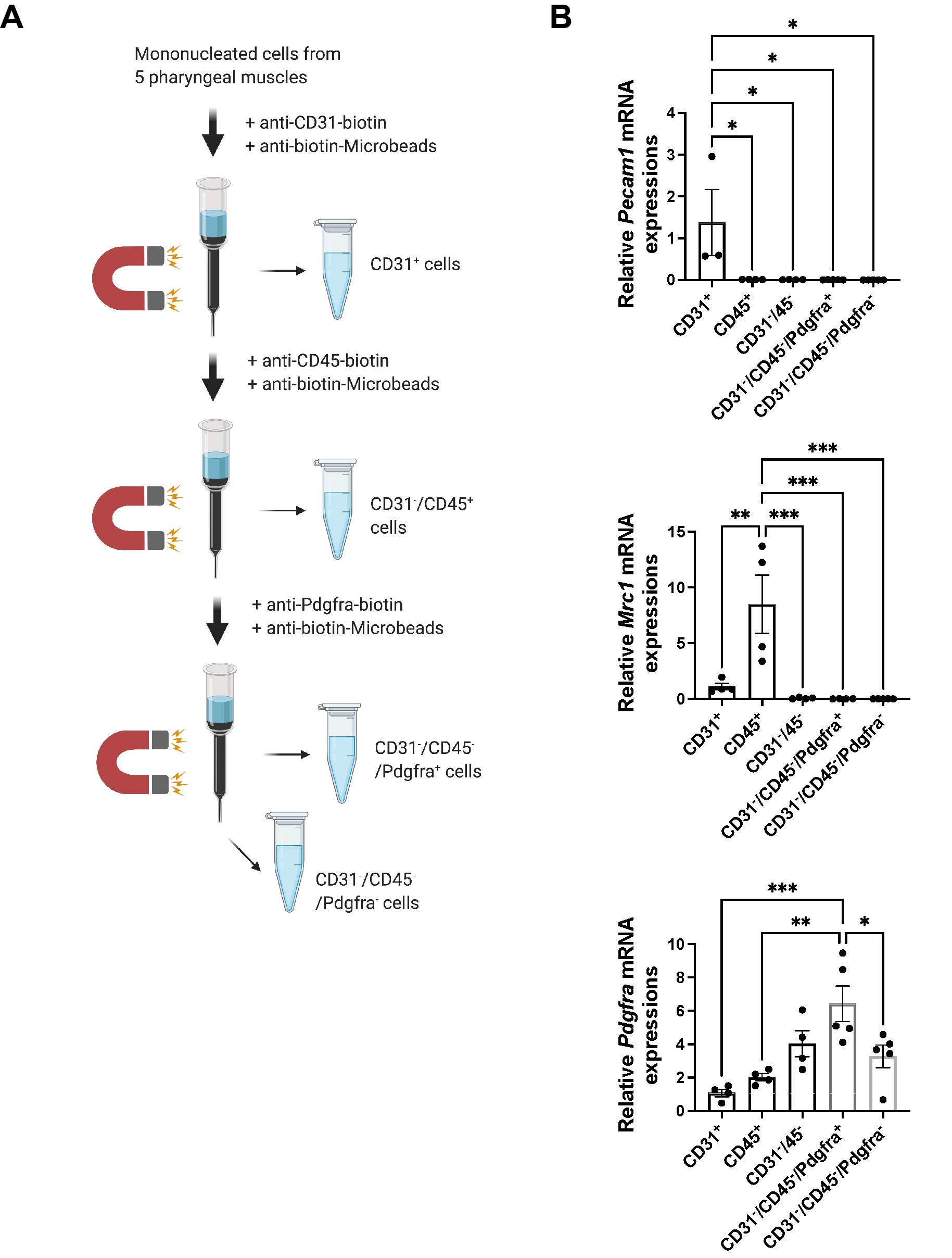

Supplement: Supplementary file 1 [file Image3.JPEG]

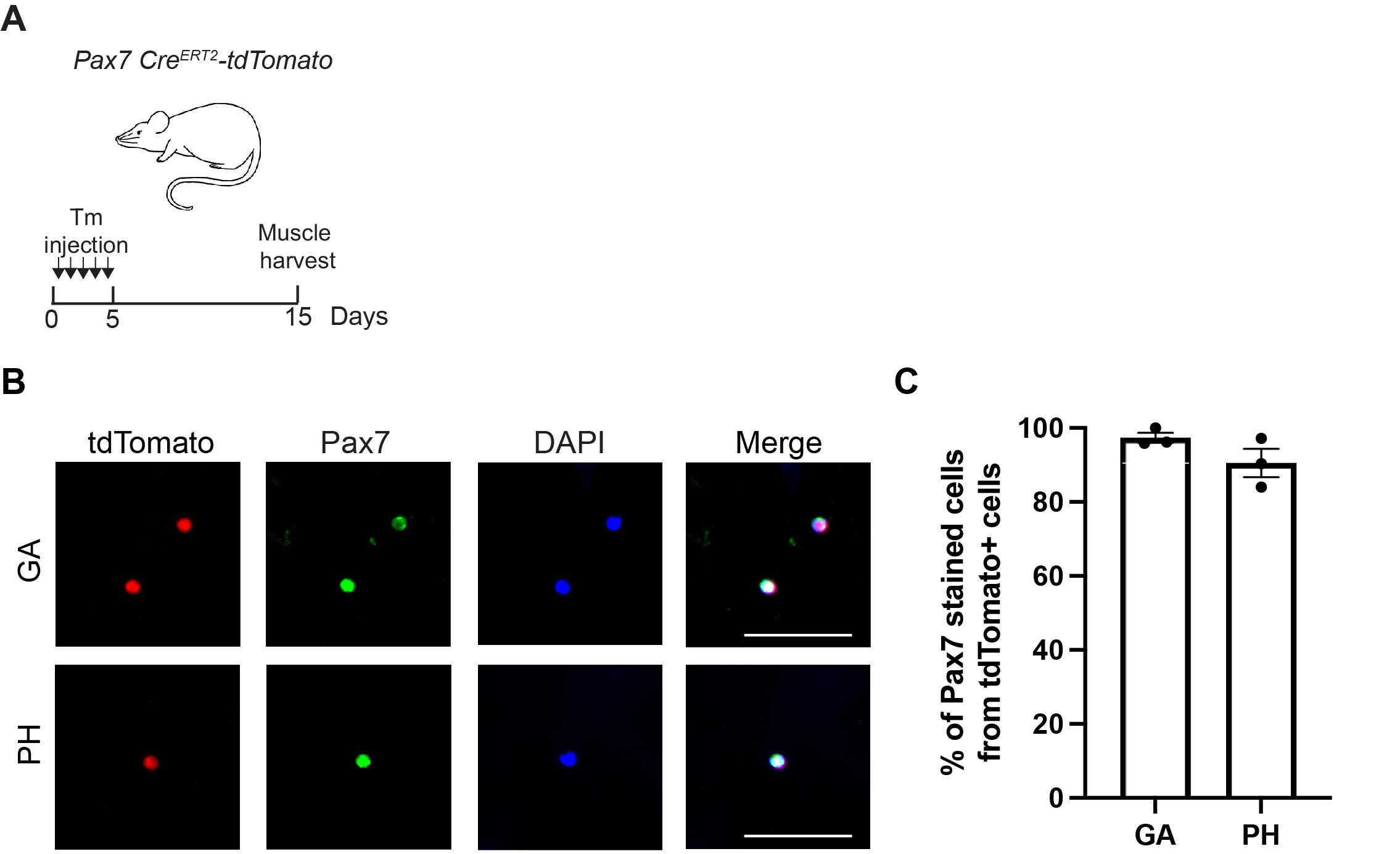

Supplement: Supplementary file 2 [file Image1.JPEG]

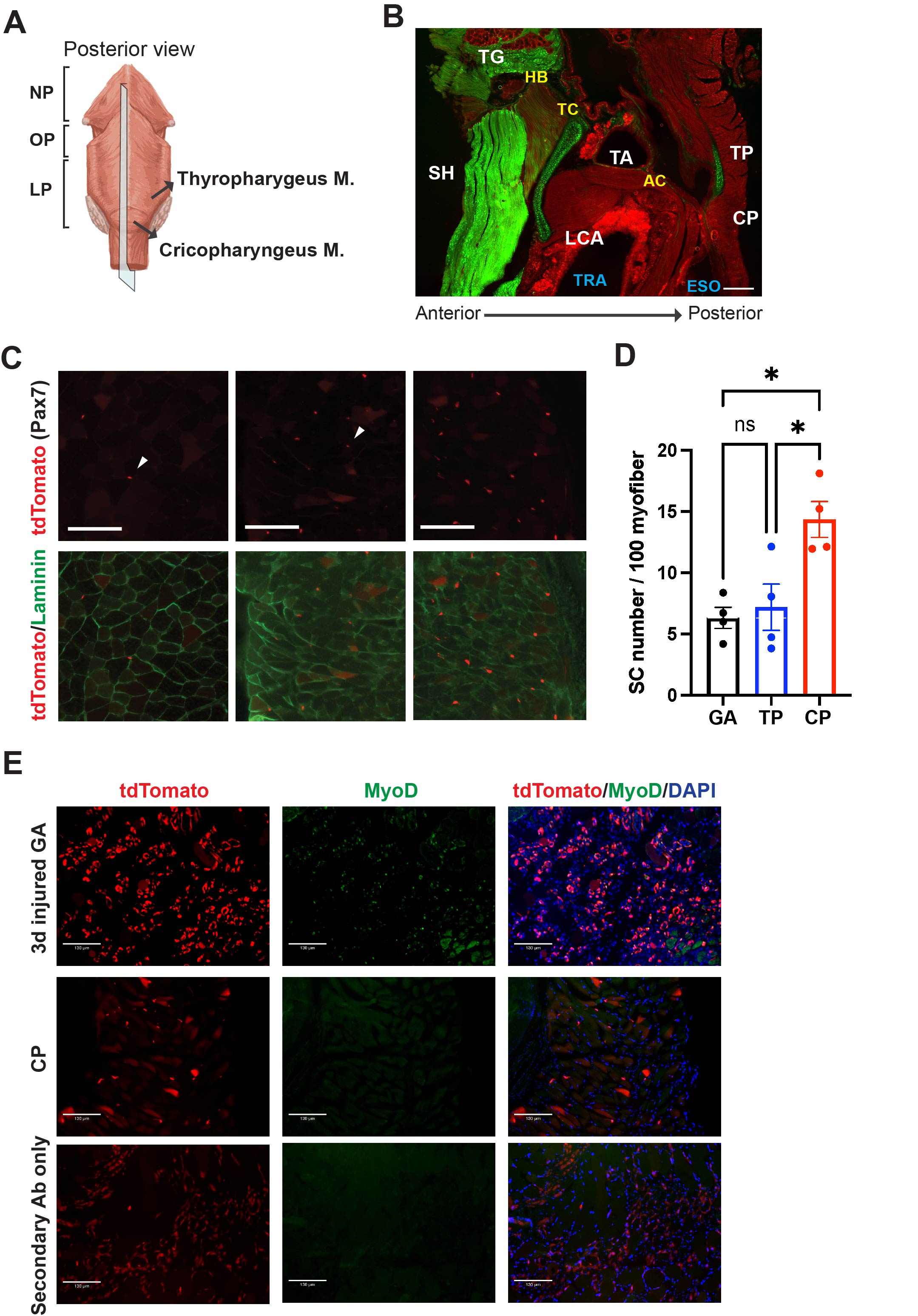

Supplement: Supplementary file 3 [file Image4.JPEG]

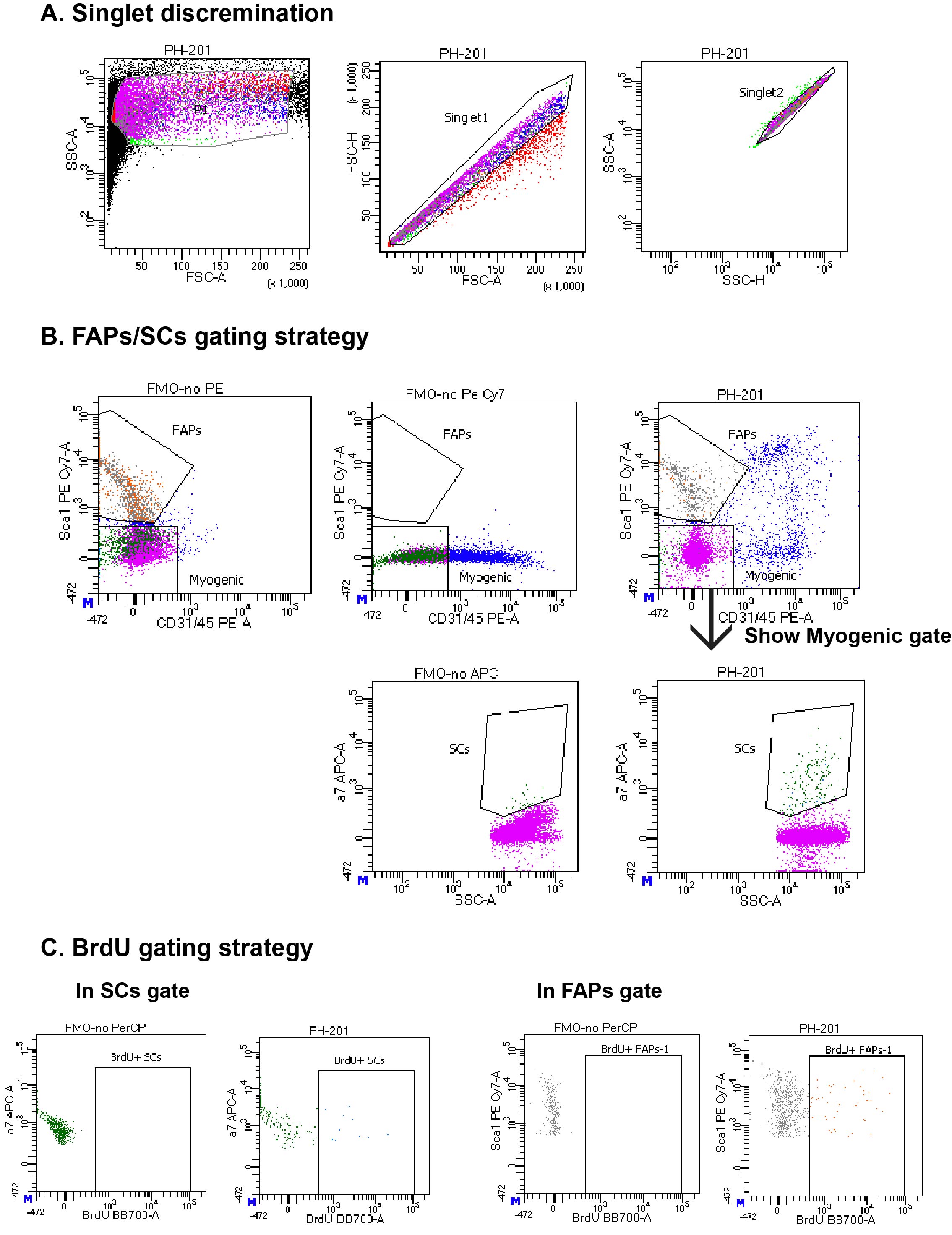

Supplement: Supplementary file 4 [file Image2.JPEG]

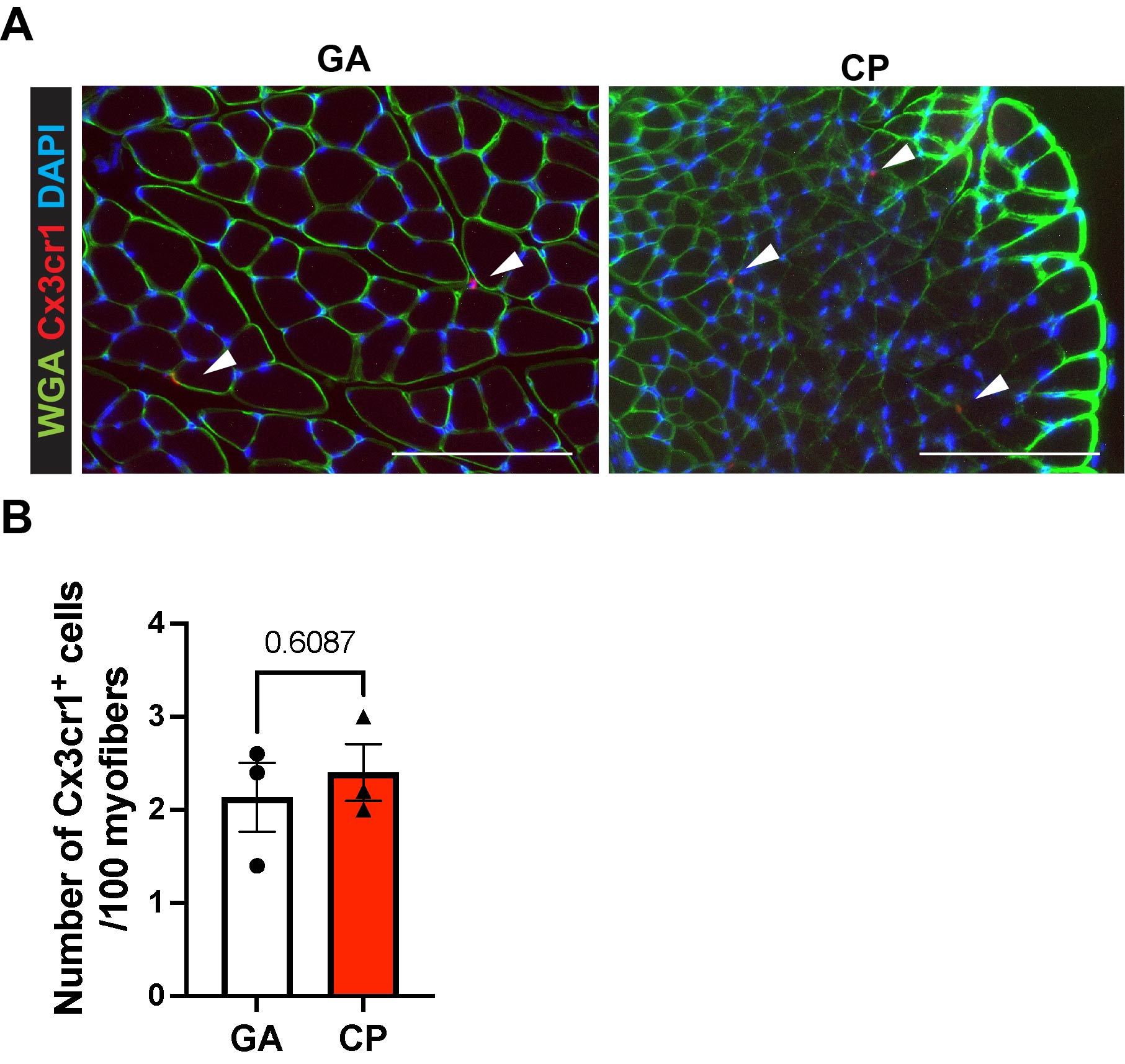

Supplement: Supplementary file 5 [file Image5.JPEG]

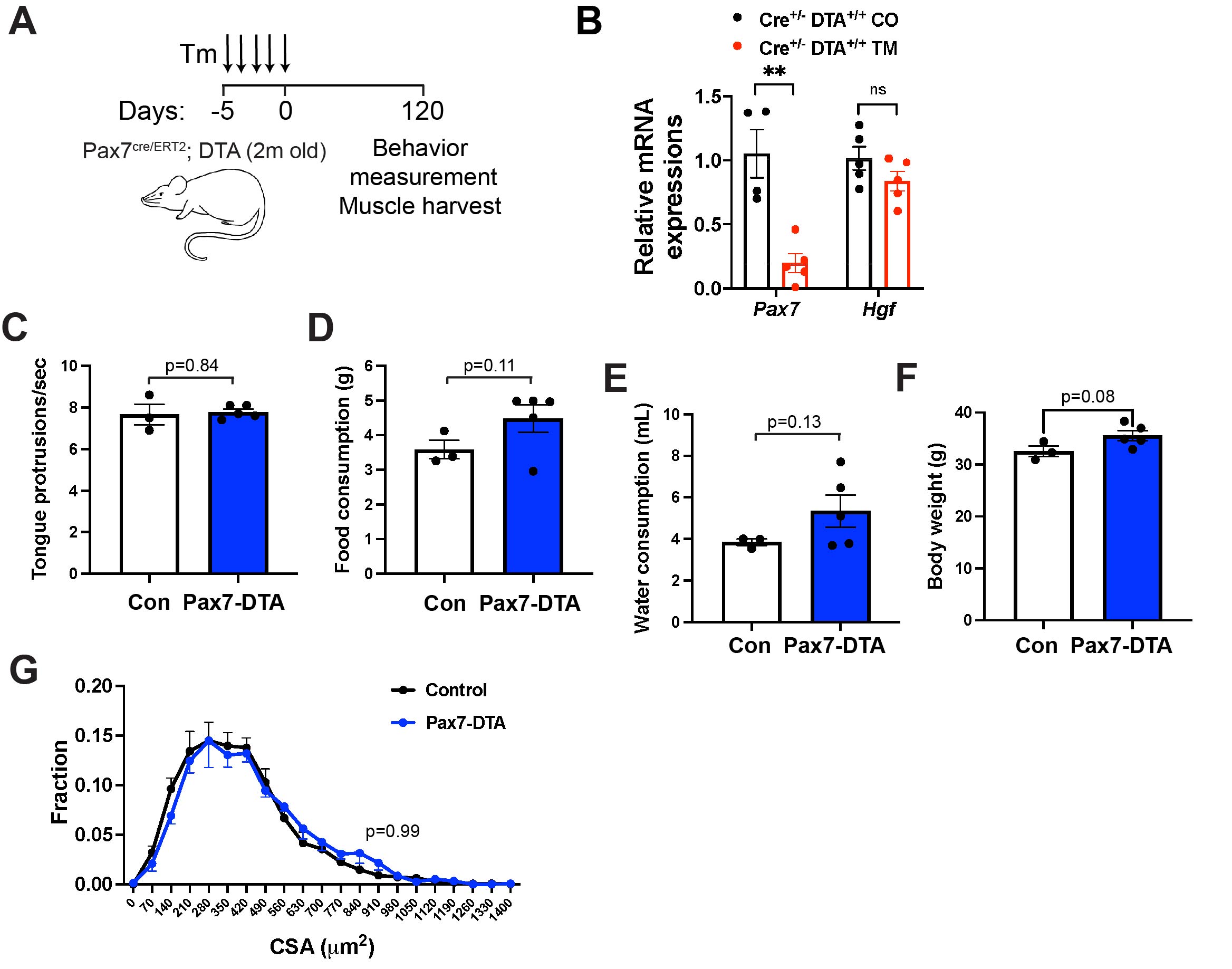

Supplement: Supplementary file 7 [file Image6.JPEG]
