# Supplementary material for: Fibroadipogenic Progenitors Regulate the Basal Proliferation of Satellite Cells and Homeostasis of Pharyngeal Muscles via HGF Secretion
Source: Front Cell Dev Biol. 2022 May 17;10:875209. doi: 10.3389/fcell.2022.875209 (PMC9164287; doi:10.3389/fcell.2022.875209)
Supplement: Supplementary file 6 [file DataSheet1.docx]

**SUPPLEMENTAL MATERIAL LEGENDS**

**Figure S1. Recombination efficiency of Pax7 Cre^ERT2^-tdTomato after tamoxifen injection.** (A) Scheme of tamoxifen injection using 3-4 months old *Pax7 Cre^ERT2^-tdTomato* mice to label SCs. (B) Representative image of cytospinned GA and PH tdTomato^+^ SC with Pax7 immunostaining to calculate recombination efficiency. Merged images show tdTomato expressed SC (Red) and immunostaining with anti-laminin antibodies (green) and DAPI (blue). Scale bars = 50 µm. (C) The averaged recombination efficiency of GA and PH tdTomato^+^ SC. n = 3.

**Figure S2. Flow cytometry singlet discrimination and gating strategy with fluorescence minus one (FMO).** (A) Representative image of SSC-A vs FSC-A scatter plot to set parental gate (P1, left panel). From the P1 gate, singlet 1 will be determined by equal values between FSC-H vs FSC-A (Singlet 1, middle panel). From Singlet 1 gate, singlet 2 will be determined by equal values between SSC-H vs SSC-A (Singlet 2, right panel). (B) FAP and SC gating strategy. From Single 2 population, FAPs gate was determined by CD31^-^/CD45^-^/Sca1^+^ cells (FAPs in the top right panel) using CD31/CD45 (PE)-FMO and Sca1 (PE-Cy7)-FMO. Similarly, myogenic gate was determined by CD31^-^/CD45^-^/Sca1^-^ cells (Myogenic in the top right panel). From the myogenic gate, SCs gate was determined by CD31^-^/CD45^-^/Sca1^-^/Integrin a7^+^ cells (Bottom right panel) using Integrin a7^+^ (APC)- FMO. (C) BrdU gating strategy. Using BrdU (PerCP)-FMO, BrdU^+^ gate was determined from SCs gate or FAPs gate.

**Figure S3. Validation of the MACS-sorted mononucleated cells in pharyngeal muscles.** (A) Scheme of experiments using MACS. Heterogenous mononucleated cell populations in pharyngeal muscle tissues. Each cell was defined by known surface protein expression. CD31 and CD45 were used to distinguish endothelial cells and immune cells, respectively. CD31^-^/CD45^-^/Sca1^+^ cells were defined as fibroadipose progenitor cells (FAPs). (B) Relative mRNA expression to validate the purity of sorted cells. *Pecam1*, *Mrc1* and *Pdgfra* genes were used as marker genes of endothelial cells, M2 macrophages and fibroadipose progenitor cells, respectively. n = 4 or 5. Statistical significance was determined by 1-way ANOVA. Asterisks indicate statistical significance (*p<0.05, **p<0.01 and ***p<0.001).

**Figure 4. Increased satellite cells in cricopharyngeal muscles.** (A) Illustration of the outer skeletal muscles surrounding the nasopharynx (NP), oropharynx (OP) and laryngopharynx (LP). (B) Embryonic origin of pharyngeal muscles. Representative longitudinal section of larynx and pharynx expressing PAX3 lineage-derived muscles (green) and non-PAX3 lineage-derived muscles (red) from 20 weeks old *Pax3^Cre/+^/mTmG* mice. Abbreviations: thyropharyngeus (TP); cricopharyngeus (CP); esophagus (ESO); trachea (TRA); tongue (TG); sternohyoid (SH); hyoid bone (HB); thyroid cartilage (TC); thyroarytenoid (TA); arytenoid cartilage (AC); lateral cricoarythenoid (LCA). Scale bars = 330 µm. (C) Representative cross-section expressing PAX7^+^ SCs (red) in gastrocnemius (GA), thyropharyngeus (TP) and cricopharyngeus (CP) muscles from 3 months old *Pax7 Cre^ERT2^-tdTomato* mouse. White arrowheads indicate examples of PAX7^+^ tdTomato expressing SCs. Basal lamina was immunostained with anti-Laminin antibodies (green). Scale bars = 130 µm. (D) Quantified numbers of SCs per 100 myofibers in gastrocnemius and pharyngeal muscles from 3 months old *Pax7 Cre^ERT2^-tdTomato* mouse. n = 4. Data were analyzed by 1-way ANOVA. For all graphs, the value represents mean ± SEM. Asterisks indicate statistical significance (ns; not significant statistically, *p<0.05, **p<0.01). (E) Representative images of MyoD immunostaining (green) on cross-sections of 3-day injured GA muscles (control) and CP muscles from *Pax7 Cre^ERT2^-tdTomato* mice. n=3. Scale bars = 130 µm.

**Figure S5. Similar levels of infiltrating macrophages between gastrocnemius and cricopharyngeal muscles.** (A) Representative cross-section expressing Cx3cr1^+^ macrophages (red) in gastrocnemius (GA) and cricopharyngeus (CP) muscles from 3 months old *cx3cr1 Cre^ERT2^-tdTomato* mouse. White arrowheads indicate examples of Cx3cr1^+^ tdTomato expressing macrophages. Muscle membrane was stained with WGA (green). Scale bars = 130 µm. (B) Quantified numbers of Cx3cr1^+^ macrophages per 100 myofibers in gastrocnemius and pharyngeal muscles from 3 months old *Pax7 Cre^ERT2^-tdTomato* mouse. n = 3. Data were analyzed by Student t-test. For all graphs, the value represents mean ± SEM. P-value was shown on the line on top of the graphs.

**Figure S6. SCs are dispensable for pharyngeal muscle function and maintenance.** (A) Scheme of strategy for satellite cell ablation using Pax7*^Cre/ERT2+/-^*-DTA (Pax7-DTA, n=4) and Pax7*^Cre/ERT2-/-^*-DTA (sex- and age-matched control, n=3). Both groups were injected with Tamoxifen (Tm). We used Pax7*^Cre/ERT2^*-DTA mice after 4 months of Tm injection. (B) Relative mRNA expression level of *Pax7*and *Hgf* in pharyngeal muscles obtained from *Pax7 Cre^ERT2^ -DTA* mice with corn oil (*Pax7 Cre^+/-^;DTA^+/+^* CO) or with tamoxifen (*Pax7 Cre^+/-^;DTA^+/+^* TM) treatment. (C) The number of tongue protrusions per second was counted when a mouse lick the water sipper using video analysis. (D-E) Each mouse was housed in a single cage to measure daily food (gram) and water (milliliter) consumption. (F) Bodyweight was measured at 4 months of Tm injection. (G) The cross-sectional area of CP muscle fibers was measured between SC-ablated mice and control mice. Statistical significance was determined by 2-way ANOVA and Sidak’s multiple comparison test (B), by Student’s t-test (C, D, F), by Mann-Whitney test (E), or multiple unpaired t-tests for each size (G). The value represents mean ± SEM. Asterisks indicate statistical significance (*p<0.05, **p<0.01).

**Table S1. Primers used for gene expression analysis**

| Genes | Primer sequences |
| --- | --- |
| *Hgf* | 5’- AAAGGGACGGTATCCATCACT -3’  5’- GCGATAGCTCGAAGGCAAAAAG -3’ |
| *Pax7* | 5’- CTGTGCTGGGACTTCTTCCT -3’  5’- AGACTCAGGGCTTGGGAAGG -3’ |
| *Plau* | 5’- GCGCCTTGGTGGTGAAAAAC -3’  5’- TTGTAGGACACGCATACACCT -3’ |
| *Plat* | 5’- GCGCCTTGGTGGTGAAAAAC -3’  5’- TTGTAGGACACGCATACACCT -3’ |
| *Hgfac* | 5’- TTCTGACCTGCTCTACCAGGAG -3’  5’- CGTTGTCCTTCACCACATAGCAC -3’ |
| *Acta1* | 5’- CCCAAAGCTAACCGGGAGAAG -3’  5’-CCAGAATCCAACACGATGCC -3’ |
| *Pecam1* | 5’-ACGCTGGTGCTCTATGCAAG -3’  5’-TCAGTTGCTGCCCATTCATCA -3’ |
| *Mrc1* | 5’- CTCTGTTCAGCTATTGGACGC -3’  5’- CGGAATTTCTGGGATTCAGCTTC -3’ |
| *Pdgfrα* | 5’- TCCATGCTAGACTCAGAAGTCA -3’  5’- TCCCGGTGGACACAATTTTTC -3’ |
| *Hprt*  *(housekeeping)* | 5’-TCAGTCAACGGGGGACATAAA -3’  5’- GGGGCTGTACTGCTTAACCAG -3’ |
| *human-PDGFRA* | 5’- CAGGGGAAACGATTGTGGTC -3’  5’- GCATTGTGATGCCTTTGCCTT -3’ |
| *human-HGF* | 5’- GCTATCGGGGTAAAGACCTACA -3’  5’- CGTAGCGTACCTCTGGATTGC -3’ |
| *human-PLAU* | 5’- TGCTCACCACAACGACATTG -3’  5’- GTGATCTCACAGCTTGTGCC -3’ |
| *human-PLAT1* | 5’- GGCCTTGTCTCCTTTCTATTCG -3’  5’ - AGCGGCTGGATGGGTACAG -3’ |
| *human-RPLP0 (housekeeping)* | 5’- ACAGACACTGGCAACATTGCGG -3’  5’- TGGTCATCCAGCAGGTGTTCGA -3’ |
|  |  |
|  |  |

**Table S2. Antibodies used for immunofluorescence staining and western blotting**

| **Detection of** | **Name** | **Host species** | **Dilution or Concentration** | **Manufacturer**  **(Cat #)** |
| --- | --- | --- | --- | --- |
| Fibroadipogenic progenitors (FAPs) | PDGFRα | Rabbit IgG | 1:200 | Cell Signaling Tech. (3174S) |
| Resident M2 macrophage | Mannose Receptor (CD206) | Rabbit IgG | 1:200 | Abcam (ab125028) |
| Basement membranes | Wheat Germ Agglutinin (WGA) | Alexa Fluor^TM^ 647 Conjugate | 1:400 | Invitrogen (W32466) |
| Basement membranes | Mouse laminin | Rabbit IgG | 1:400 | Sigma (L9393) |
| Basement membranes | Human dystrophin (Dyst) | Mouse IgG | 1:200 | Sigma (D8168) |
| Hepatocyte Growth Factor | HGF | Mouse IgG2a | 1:2000 | Novus Biologicals (NBP1-19182) |
| glyceraldehyde-3-phosphate dehydrogenase | GAPDH | Rabbit IgG | 1:10000 | Bethyl Laboratories (A300-639A) |
| SC activation | MyoD | Rabbit IgG | 1:50 | Santa Cruz Biotechnology  (sc-32758) |
| Human fibroadipogenic progenitors (FAPs) | CD90 | Mouse IgG1 | 1:50 | BD Pharmingen (555593) |
| Basement membranes | Human laminin | Rabbit IgG | 1:300 | Dako (Z0097) |
